# Supplementary material for: Automating multi-label crisis detection in psychological support hotlines with pre-trained models
Source: PLOS Digit Health. 2026 May 13;5(5):e0001383. doi: 10.1371/journal.pdig.0001383 (PMC13170875; doi:10.1371/journal.pdig.0001383)
Supplement: S1 Appendix — (DOCX) [file pdig.0001383.s001.docx]

**S1 Appendix. Human expert evaluation dimensions**

We used GPT to generate natural language explanations based on the call text and the model's predictions of the caller's emotional state (depressed vs. normal), suicidal ideation (yes vs. no), suicide plan (yes vs. no), and overall high-risk status of callers (high risk vs. non-high risk). We conducted human expert evaluations from five dimensions: consistency with labeling, logic and reasoning plausibility, completeness and accuracy, clinical and situational relevance, clarity and comprehensibility. The following is a detailed description of the evaluation indicators.

1. **Consistency with labeling:**

Does the generated explanation accurately reflect the model's predicted labels (note that this refers to labels such as "depression," "suicidal ideation," "suicidal plan," and "high risk," rather than your own judgment)? Does the explanation reasonably establish the relationship between the content of the call and these labels?

1. **Logic and reasoning plausibility:**

How logical is the explanation? Is the reasoning process rational, and does it clearly demonstrate how the labels are derived from the call content? Lack of clarity or insufficient reasoning may undermine the credibility of the explanation.

1. **Completeness and accuracy:**

Does the explanation include all necessary information? Are there any omissions of critical content or inclusion of erroneous information? Ensuring the completeness and accuracy of the explanation is critical for clinical decision-making.

1. **Clinical and situational relevance:**

Is the explanation practically applicable in a clinical context? Does it correlate with the caller's actual emotional state and suicide risk assessment? This involves determining whether the explanation can effectively guide professionals in making appropriate decisions in real-world scenarios.

1. **Clarity and comprehensibility:**

Is the generated explanation clear and easy to understand? Can it be quickly comprehended and applied by professionals? In practical settings, the clarity and comprehensibility of the explanation are of paramount importance.
