# Supplementary material for: Automating multi-label crisis detection in psychological support hotlines with pre-trained models
Source: PLOS Digit Health. 2026 May 13;5(5):e0001383. doi: 10.1371/journal.pdig.0001383 (PMC13170875; doi:10.1371/journal.pdig.0001383)
Supplement: S1 Fig — (DOCX) [file pdig.0001383.s002.docx]

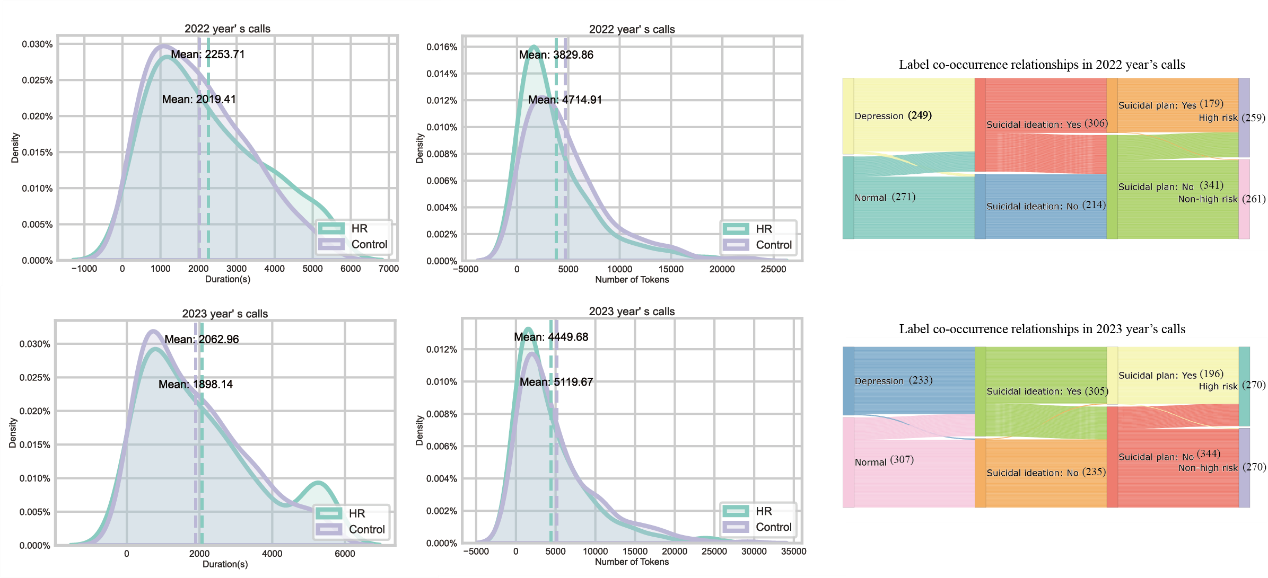


**S1 Fig.** Statistics information of high-risk and control group calls in 2022 and 2023.

The distribution of call durations included in the study for 2022 and 2023 (as shown in S1 Fig) is represented by KDE curves. In 2022, the mean call duration for the high-risk group was 2,253 s, compared to 2,091 s for the control group. An independent samples t-test was used to compare the distributions, and no statistically significant difference was found between the two groups. In 2023, the mean call duration for the high-risk group was 2,062 s, compared to 1,988 s for the control group, and similarly, an independent samples t-test revealed no statistically significant difference in the distribution between the two groups. When comparing the distributions across the two years, the duration distributions of both groups were broadly similar. Overall, high-risk calls tended to have longer durations than control group calls, which may suggest that high-risk callers faced more complex situations, requiring more careful listening and guidance. The distribution of token counts from the callers' texts in 2022 and 2023 is also shown in S1 Fig. In 2022, the high-risk group had a mean of 8,829 tokens, compared to 8,744 for the control group. In 2023, the means were 9,449 and 9,119, respectively. Each call recording has four binary labels: mood status (depression vs. normal), suicidal ideation (yes vs. no), suicide plan (yes vs. no), and high-risk vs. non-high-risk. There is a certain interdependent internal relationship between these labels. S1 Fig further illustrates the co-occurrence relationship of call labels in different years.
