# Supplementary material for: Automating multi-label crisis detection in psychological support hotlines with pre-trained models
Source: PLOS Digit Health. 2026 May 13;5(5):e0001383. doi: 10.1371/journal.pdig.0001383 (PMC13170875; doi:10.1371/journal.pdig.0001383)
Supplement: S2 Fig — (DOCX) [file pdig.0001383.s003.docx]

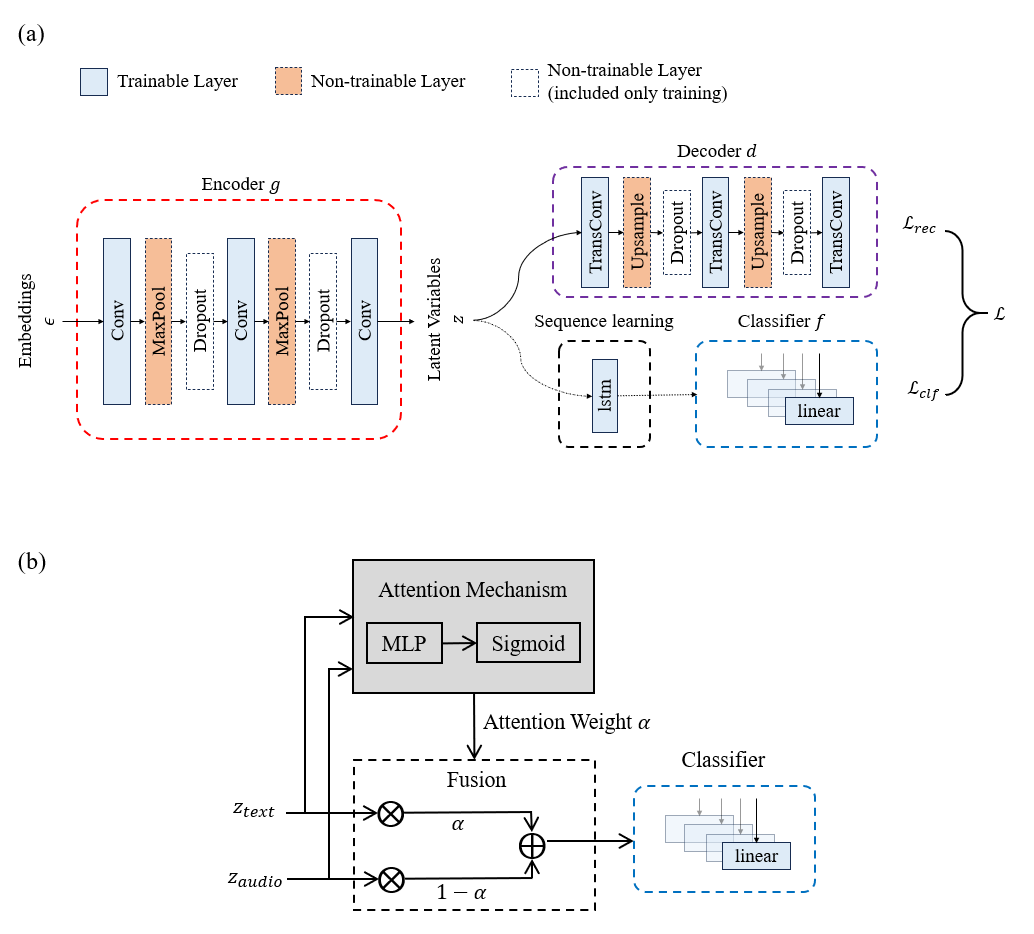


**S2 Fig.** The neural network classification framework.

We designed a neural network classification framework for the text vectors generated from hotline call transcriptions (as shown in S2 Fig (a)). By combining the reconstruction task of an autoencoder with the classification task, we maximized the utilization of input data features for multi-label classification. An autoencoder is a type of artificial neural network used for unsupervised learning, typically aimed at learning a compressed, efficient representation of data. It consists of two main parts: the encoder, which maps the input data to a lower-dimensional latent space, and the decoder, which attempts to reconstruct the original input from this compressed representation. In our network structure, the encoder is composed of multiple convolutional layers (Conv) and max-pooling layers (MaxPool). The MaxPool layer also includes Dropout to prevent overfitting. The output from the encoder is the latent variable $z$, which contains high-level feature representations extracted by the encoder. The latent variable $z$ is then fed into the decoder, which is composed of transposed convolutional layers (TransConv), upsampling layers (Upsample), and Dropout, attempting to reconstruct the input embeddings.

When the input $\epsilon$ is a two-dimensional matrix sequence​, the latent variable $z$ is first fed into the LSTM module, which captures the sequential features of the text and addresses the issue of sequential dependencies in the text. The output of the LSTM module is passed through a linear layer to finally produce the prediction results $\hat{y}$​ for the four binary classification labels. When the input $\epsilon$ is a one-dimensional vector, the latent variable $z$ is directly fed into the linear classification layer to obtain the prediction result $\hat{y}_{i}^{(j)}$​.

S2 Fig (b) illustrates the Attention-based Gated Fusion architecture used to fuse audio and text input. The latent representations from the audio ${(z}_{audio})$ and text ${(z}_{text})$encoders are first concatenated to form a joint context vector. This vector is passed through a Multi-Layer Perceptron (MLP) consisting of a 64-unit hidden layer with ReLU activation and a single-unit output layer with Sigmoid activation. The network outputs a scalar attention weight $\alpha\in[0, 1]$. This weight dynamically modulates the contribution of the audio stream, while its complement $(1-\alpha)$ modulates the text stream, enabling adaptive multimodal fusion.
