# Supplementary material for: Automating multi-label crisis detection in psychological support hotlines with pre-trained models
Source: PLOS Digit Health. 2026 May 13;5(5):e0001383. doi: 10.1371/journal.pdig.0001383 (PMC13170875; doi:10.1371/journal.pdig.0001383)
Supplement: S3 Fig — (DOCX) [file pdig.0001383.s004.docx]

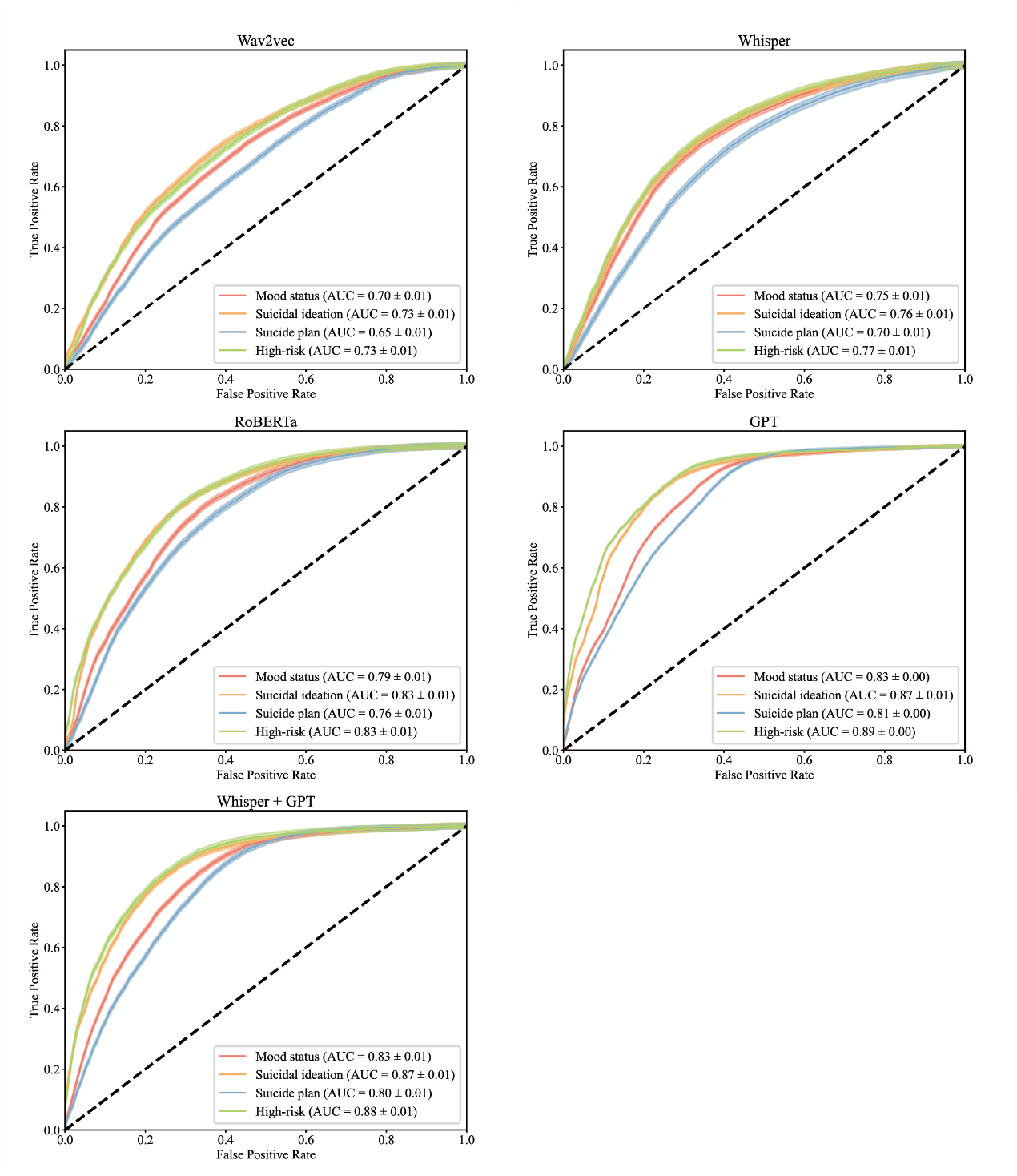


**S3 Fig.** ROC Curves for the multidimensional prediction with crafted models.

S3 Fig shows the Receiver Operating Characteristic (ROC) curves of the multidimensional deep learning classification model using different pre-trained models. The dark curve represents the mean of the model evaluation metric in the forward test set after 100 iterations, and the light shade represents the standard deviation.
