# Supplementary material for: Automating multi-label crisis detection in psychological support hotlines with pre-trained models
Source: PLOS Digit Health. 2026 May 13;5(5):e0001383. doi: 10.1371/journal.pdig.0001383 (PMC13170875; doi:10.1371/journal.pdig.0001383)
Supplement: S7 Fig — (DOCX) [file pdig.0001383.s008.docx]

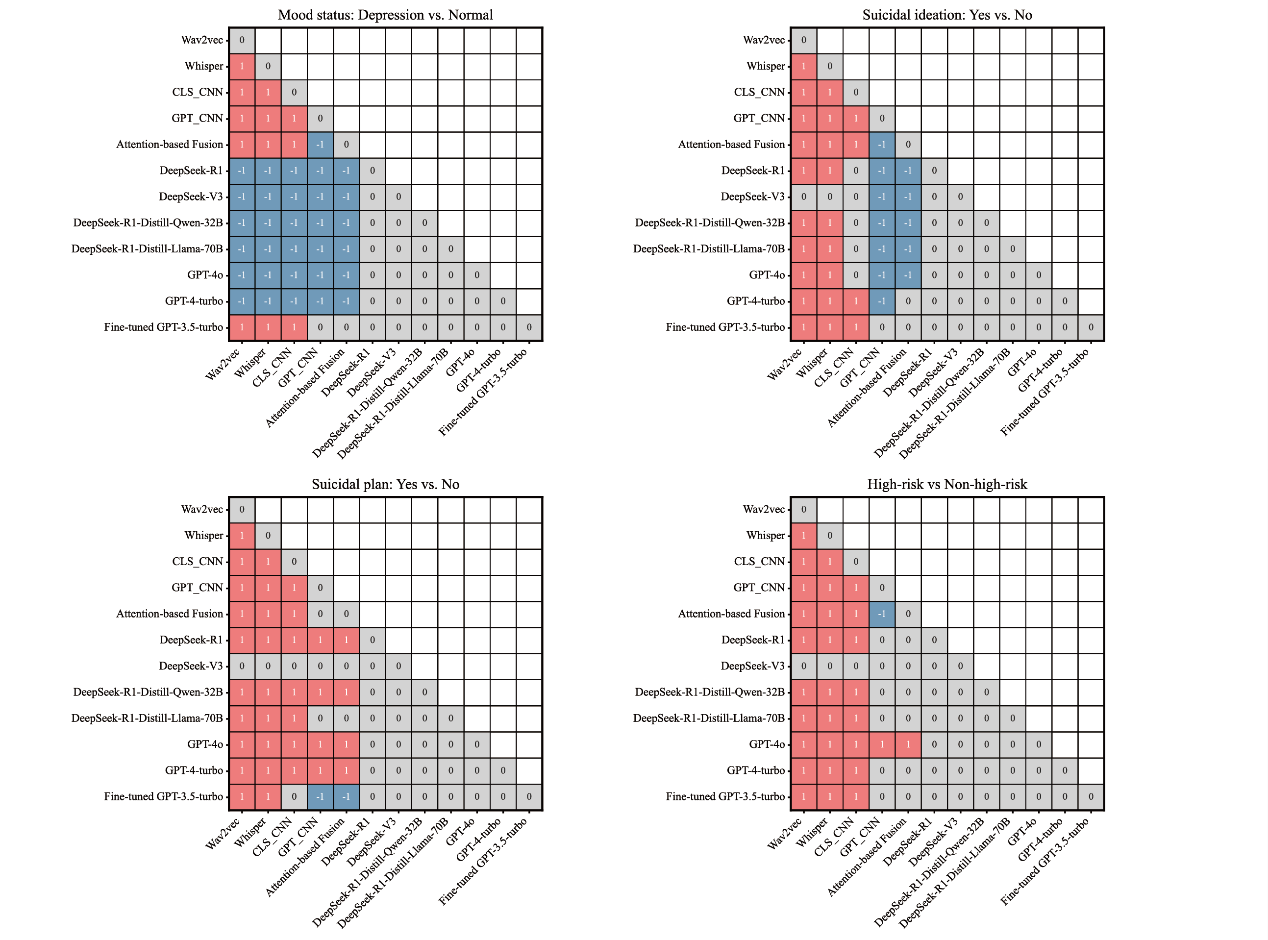


**S7 Fig.** Statistical Comparison of Multi-Dimensional Prediction Methods Based on Accuracy.

Figure S7 shows the statistical comparative analysis of the accuracies obtained from various methodologies employed for multi-dimensional prediction. Within the deep learning classification framework utilizing pre-trained models, the audio pre-trained models Wav2vec 2.0 and Whisper-small, which demonstrated superior performance, were selected for comparison. This comparison also included the text-processing models like RoBERTa and GPT embedding models. In the strategy of LLM-based prediction via prompt engineering, the models encompassed OpenAI’s GPT-4 series models (GPT-4o and GPT-4-turbo) and the DeepSeek series models (DeepSeek-R1, DeepSeek-V3, DeepSeek-R1-Distill-Qwen-32B, DeepSeek-R1-Distill-Llama-70B).

Significance was determined using a rigorous statistical comparison framework: Welch’s t-test was used for large-scale (100 vs. 100) model comparisons; Permutation tests were employed for mixed-scale (100 vs. 5) comparisons; and the Wilcoxon Rank Sum test was applied for small-scale (5 vs. 5) comparisons, followed by Holm-Bonferroni correction to control for multiple comparisons. In the figure, a value of “1” indicates that the metric performance of the model on the vertical axis is significantly higher than that of the model on the horizontal axis ($p$<0.05 after correction), a value of “-1” indicates that the metric performance of the model on the vertical axis is significantly lower than that of the model on the horizontal axis ($p$<0.05 after correction), and a value of “0” indicates no significant difference.
