# Supplementary material for: Automating multi-label crisis detection in psychological support hotlines with pre-trained models
Source: PLOS Digit Health. 2026 May 13;5(5):e0001383. doi: 10.1371/journal.pdig.0001383 (PMC13170875; doi:10.1371/journal.pdig.0001383)
Supplement: S8 Fig — (DOCX) [file pdig.0001383.s009.docx]

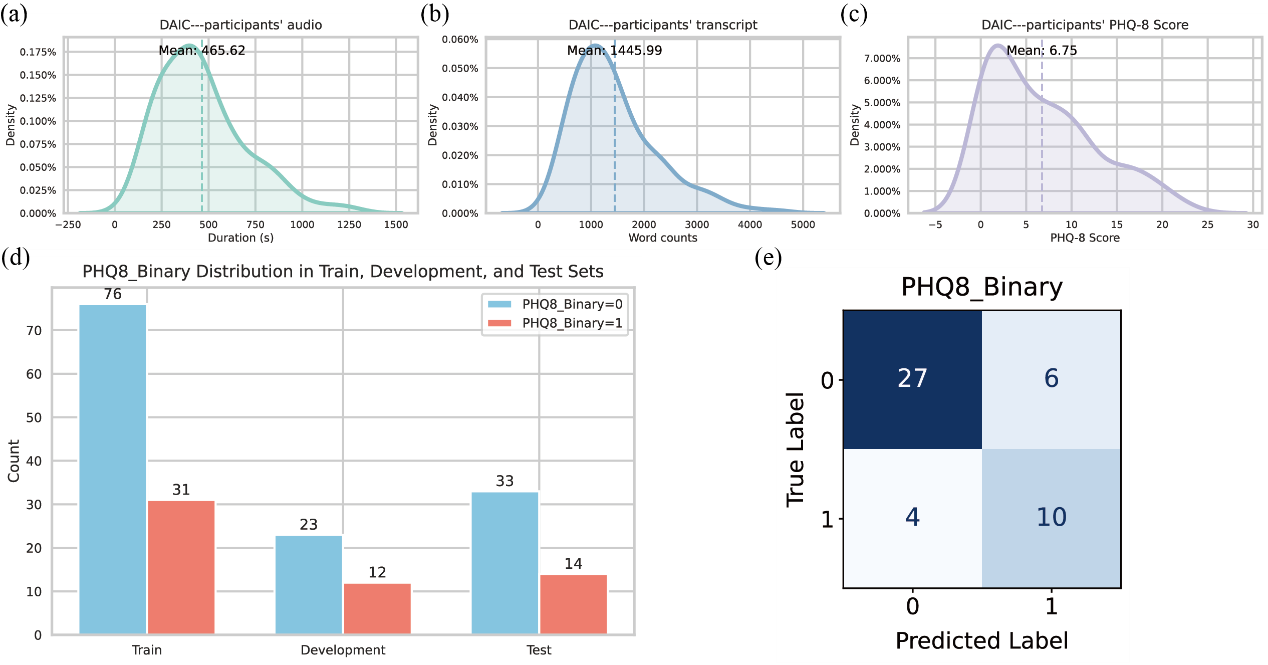


**S8 Fig.** Cross-linguistic generalizability validation using the Distress Analysis Interview Corpus.

S8 Fig illustrates the data characteristics and classification performance of the cross-linguistic transfer experiment conducted on the English-language Distress Analysis Interview Corpus (DAIC) dataset. (a–c) Dataset Distributions: These panels display the density distributions for the DAIC participants' (a) audio duration in seconds, (b) transcript word counts, and (c) clinical depression severity scores based on the PHQ-8. (d) Data Split Composition: The bar chart shows the binary distribution of the PHQ-8 depression labels (0: non-depressed, 1: depressed) across the Training, Development, and Test sets, involving a total of 189 participants. (e) Classification Performance: The confusion matrix presents the results of the GPT-embedding-based framework combined with an SVM classifier on the DAIC test set ($n=47$).
