# Supplementary material for: Automating multi-label crisis detection in psychological support hotlines with pre-trained models
Source: PLOS Digit Health. 2026 May 13;5(5):e0001383. doi: 10.1371/journal.pdig.0001383 (PMC13170875; doi:10.1371/journal.pdig.0001383)
Supplement: S1 Table — (DOCX) [file pdig.0001383.s010.docx]

**S1 Table.** Depression and Suicide Risk Assessment Questionnaire

| **Date of call** | | Time | **Number** | Caller ID | **Counselor** | | Name |
| --- | --- | --- | --- | --- | --- | --- | --- |
| **Depression Assessment:** No depression/ Mild depression /Moderate depression/ Major depression | | | | | | | |
| No. | Question | | | | | Answer | |
| 1 | Have you felt down or depressed recently? (sad face, sighing, tears alone) | | | | | Yes (duration) | |
|  |  |  |  |  |  | No | |
| 2 | Have you recently lost interest in things you normally enjoy? | | | | | Yes (duration) | |
|  |  |  |  |  |  | No | |
| 3 | Have you recently felt worthless, a useless person or a failure? | | | | | Yes | |
|  |  |  |  |  |  | No | |
| 4 | Do you blame yourself or have regrets? | | | | | Yes | |
|  |  |  |  |  |  | No | |
| 5 | Has your weight changed recently? | | | | | Yes  (increase decrease) | |
|  |  |  |  |  |  | No | |
|  | Any recent changes in your appetite? | | | | | Yes  (increase decrease) | |
|  |  |  |  |  |  | No | |
| 6 | Has your sleep changed recently? | | | | | Yes  (increase decrease) | |
|  |  |  |  |  |  | No | |
| 7 | Have you had any physical complaints recently? (Dizziness, fatigue, listlessness or lack of energy) | | | | | Yes | |
|  |  |  |  |  |  | No | |
| 8 | Have you recently had trouble concentrating, thinking flexibly, or being stupid? | | | | | Yes | |
|  |  |  |  |  |  | No | |
| 9 | Did others see that you were different than usual (expression, speech, behavior, reaction)? | | | | | Yes | |
|  |  |  |  |  |  | No | |
| 10 | Has the above problem affected your work, study, life, socialization, etc.? | | | | | Yes | |
|  |  |  |  |  |  | No | |
| 11 | Do you feel distressed when these problems arise? | | | | | Yes | |
|  |  |  |  |  |  | No | |
| 12 | Many people have thought about death when they are in the above situations; have you ever thought about harming yourself? | | | | | Yes | |
|  |  |  |  |  |  | No | |
| **Suicide Risk Assessment:** High-risk/ Non-high-risk | | | | | | | |
| 13 | Do you have a specific plan for self-injury or suicide? | | | | | Yes (describe: ) | |
|  |  |  |  |  |  | No | |
|  | What is the intended time frame for this program? | | | | | Yes (describe: ) | |
|  |  |  |  |  |  | No | |
|  | Do you really want to die? | | | | | Yes | |
|  |  |  |  |  |  | No | |
| 14 | Have you ever committed suicide or intentionally harmed yourself before? | | | | | Yes (describe: ) | |
|  |  |  |  |  |  | No | |
|  | How many suicidal behaviors were there? | | | | | Times. | |
|  | When did this last happen? | | | | | Y/M/D | |
|  | Did you really want to die at that time? | | | | | Yes | |
|  |  |  |  |  |  | No | |
| 15 | Have any of your relatives, friends, co-workers, or acquaintances ever self-injured or committed suicide? | | | | | Yes | |
|  |  |  |  |  |  | No | |
| 16 | If "0" means no hope and "100" means the most hope, how hopeful are you about your future life? | | | | | ≤50 | |
|  |  |  |  |  |  | >50 | |
| 17 | In the past month, have you had any serious physical illnesses, major life events? | | | | | Yes | |
|  |  |  |  |  |  | No | |
| 18 | In the last month, have you consumed excessive alcohol, abused sleeping pills, narcotics or stimulants? | | | | | Yes | |
|  |  |  |  |  |  | No | |
| 19 | What are the main causes of your suicidal thoughts? | | | | |  | |
| 20 | What problem do you hope to solve or achieve through suicide? | | | | |  | |
| **Depression and Suicide Risk Assessment Criteria** | | | | | | | |
| No depression: Items 1 and 2 answered “No”, and no suicidal ideation reported; Mild depression: Either item 1 or 2 is “Yes”; 4 out of items 1–9 are “Yes”; and either item 10 (functional impairment) or item 11 (distress) is “Yes”; Moderate depression: Either item 1 or 2 is “Yes”; 5–6 of items 1–9 are “Yes”; and either item 10 or 11 is “Yes”; Major depression: Either item 1 or 2 is “Yes” for at least one week in duration; ≥7 of items 1–9 are “Yes”; and either item 10 or 11 is “Yes”.  Risk factor scoring: 1 point for “Yes” on item 14 (past suicide attempts); 1 point for “Yes” on item 15 (exposure to others’ self-harm/suicide); 1 point if the hopefulness score in item 16 is ≤50; 1 point for “Yes” on either item 17 (serious illness or major life events) or item 18 (substance abuse); 1 point for moderate depression; 2 points for severe depression.  High-risk caller: Defined as (1) reporting suicidal ideation with a specific suicide plan, or (2) reporting suicidal ideation without a plan but with a total risk factor score ≥ 5. | | | | | | | |

Note: The original questionnaire and assessment criteria are in Chinese. This table is the English translation version.
