# Supplementary material for: Automating multi-label crisis detection in psychological support hotlines with pre-trained models
Source: PLOS Digit Health. 2026 May 13;5(5):e0001383. doi: 10.1371/journal.pdig.0001383 (PMC13170875; doi:10.1371/journal.pdig.0001383)
Supplement: S2 Table — (DOCX) [file pdig.0001383.s011.docx]

**S2 Table.** The carefully crafted prompt for the multi-label prediction

| As a crisis hotline analyst, your task is to evaluate the caller’s depressive state and suicide risk based on the given call content. You can determine the suicide risk step by step by assessing:  1. Whether there are suicidal thoughts,  2. Whether a suicide plan is in place,  3. Whether there has been past suicidal behavior,  4. Whether there is any current suicidal action being taken.  The levels of suicide risk, from lowest to highest, are: 1. No suicidal thoughts, 2. Thoughts with no plan, 3. A plan but no immediate intent to act, 4. A plan with the possibility of acting soon, 5. Imminent action, 6. A suicide attempt within the last two weeks, 7. Recently attempted suicide.  The call content is provided to you in the form of a sentence list. Please analyze the depressive state and suicide risk based on the urgency of the situation. Make predictions and assign labels from four perspectives:   - Emotion status: Depressed or Normal, - Suicidal thoughts: Yes or No, - Suicide plan: Yes or No, - High risk: High risk or Non-high risk.   A “high-risk” call refers to a situation where there is an immediate and severe psychological crisis, with an urgent suicide risk that requires immediate intervention. A “low-risk” call refers to a situation where the caller’s psychological problems can be alleviated through conversation, leading to emotional stability, or where a recommendation for further professional help is appropriate.  You should output only a JSON dict exactly formatted as: dict{“Mood status”: str(), “Suicidal ideation”: str(), “Suicidal plan”: str(), “High risk vs. Non-high risk”: str()}  {few_shot_exemplar}  Here is the content of a non-high risk caller, each sentence is led by a sentence_id: {sentences}  Plain JSON output: {label_str}  Here is the content of a high-risk caller, each sentence is led by a sentence_id: {sentences}  Plain JSON output: {label_str} |
| --- |
