# Supplementary material for: Automating multi-label crisis detection in psychological support hotlines with pre-trained models
Source: PLOS Digit Health. 2026 May 13;5(5):e0001383. doi: 10.1371/journal.pdig.0001383 (PMC13170875; doi:10.1371/journal.pdig.0001383)
Supplement: S3 Table — (DOCX) [file pdig.0001383.s012.docx]

**S3 Table.** Based on the following prompt, we asked for the reasoning behind the classification

| Consider the following conversation content of a call to a psychological hotline and provide it as a list of sentences. The call content is as follows: {sentences}.  It is known that the emotional state and suicide risk label for this call are: {label_str}.  Please analyze the call content and explain the labeling, indicating the original text as evidence for your explanation. |
| --- |
