# Supplementary material for: Automating multi-label crisis detection in psychological support hotlines with pre-trained models
Source: PLOS Digit Health. 2026 May 13;5(5):e0001383. doi: 10.1371/journal.pdig.0001383 (PMC13170875; doi:10.1371/journal.pdig.0001383)
Supplement: S4 Table — (DOCX) [file pdig.0001383.s013.docx]

**S4 Table.** Performance evaluation for the multidimensional prediction with crafted models

| **Methods** | **Precision** | **Recall** | **F1-Score** | **Accuracy** |
| --- | --- | --- | --- | --- |
|  | **Mood status: Depression vs. Normal** | | | |
| Wav2vec | 0.5863  [0.5814, 0.5914] | 0.5926  [0.5680, 0.6155] | 0.5801  [0.5654, 0.5923] | 0.6415  [0.6386, 0.6442] |
| HuBERT | 0.5649  [0.5454, 0.5795] | 0.5269  [0.4874, 0.5645] | 0.5227  [0.4928, 0.5497] | 0.6256  [0.6208, 0.6303] |
| Whisper-chinese | 0.6110  [0.6032, 0.6186] | 0.6887  [0.6641, 0.7125] | 0.6380  [0.6286, 0.6465] | 0.6704  [0.6658, 0.6749] |
| Whisper-small | 0.6362  [0.6294, 0.6424] | 0.6556  [0.6309, 0.6796] | 0.6370  [0.6251, 0.6479] | 0.6862  [0.6820, 0.6902] |
| Whisper-medium | 0.5811  [0.5726, 0.5893] | 0.5859  [0.5516, 0.6210] | 0.5641  [0.5486, 0.5789] | 0.6292  [0.6231, 0.6346] |
| Whisper-large | 0.5974  [0.5900, 0.6044] | 0.6226  [0.5916, 0.6524] | 0.5944  [0.5799, 0.6079] | 0.6494  [0.6446, 0.6538] |
| RoBERTa | 0.6405  [0.6347, 0.6462] | 0.7671  [0.7498, 0.7839] | 0.6939  [0.6875, 0.6994] | 0.7114  [0.7080, 0.7146] |
| GPT embedding | **0.6864**  **[0.6827, 0.6902]** | 0.7866  [0.7765, 0.7970] | **0.7316**  **[0.7285, 0.7348]** | **0.7525**  **[0.7509, 0.7542]** |
| Attention-based Fusion | 0.6726  [0.6694, 0.6757] | **0.7997**  **[0.7922, 0.8073]** | 0.7299  [0.7270, 0.7329] | 0.7458  [0.7435, 0.7481] |
|  | **Suicidal ideation: Yes vs. No** | | | |
| Wav2vec | 0.6920  [0.6863, 0.6978] | 0.7803  [0.7617, 0.7976] | 0.7289  [0.7213, 0.7349] | 0.6763  [0.6729, 0.6792] |
| HuBERT | 0.6628  [0.6531, 0.6721] | 0.8012  [0.7746, 0.8266] | 0.7153  [0.7058, 0.7232] | 0.6466  [0.6391, 0.6537] |
| Whisper-chinese | 0.7109  [0.7041, 0.7176] | 0.7918  [0.7733, 0.8098] | 0.7443  [0.7377, 0.7503] | 0.6964  [0.6922, 0.7005] |
| Whisper-small | 0.7222  [0.7154, 0.7291] | 0.7889  [0.7709, 0.8064] | 0.7492  [0.7431, 0.7549] | 0.7052  [0.7016, 0.7086] |
| Whisper-medium | 0.6646  [0.6560, 0.6732] | 0.7948  [0.7677, 0.8209] | 0.7132  [0.7034, 0.7216] | 0.6477  [0.6433, 0.6518] |
| Whisper-large | 0.6847  [0.6764, 0.6930] | 0.7917  [0.7714, 0.8118] | 0.7273  [0.7209, 0.7330] | 0.6691  [0.6648, 0.6732] |
| RoBERTa | 0.7410  [0.7347, 0.7474] | 0.8767  [0.8648, 0.8879] | 0.8008  [0.7971, 0.8038] | 0.7546  [0.7512, 0.7578] |
| GPT embedding | **0.8043**  **[0.8004, 0.8080]** | **0.8869**  **[0.8806, 0.8932]** | **0.8429**  **[0.8410, 0.8447]** | **0.8136**  **[0.8116, 0.8155]** |
| Attention-based Fusion | 0.7921  [0.7885, 0.7955] | 0.8744  [0.8683, 0.8803] | 0.8306  [0.8284, 0.8328] | 0.7989  [0.7966, 0.8013] |
|  | **Suicidal plan: Yes vs. No** | | | |
| Wav2vec | 0.4900  [0.4713, 0.5055] | 0.2642  [0.2390, 0.2892] | 0.3293  [0.3047, 0.3524] | 0.6417  [0.6392, 0.6442] |
| HuBERT | 0.3797  [0.3382, 0.4195] | 0.1558  [0.1272, 0.1858] | 0.2009  [0.1685, 0.2343] | 0.6378  [0.6357, 0.6398] |
| Whisper-chinese | 0.5286  [0.5224, 0.5347] | 0.3813  [0.3515, 0.4109] | 0.4247  [0.4031, 0.4457] | 0.6533  [0.6505, 0.6561] |
| Whisper-small | 0.5437  [0.5374, 0.5500] | 0.3332  [0.3038, 0.3625] | 0.3927  [0.3699, 0.4151] | 0.6571  [0.6544, 0.6597] |
| Whisper-medium | 0.4277  [0.3979, 0.4585] | 0.0964  [0.0789, 0.1161] | 0.1422  [0.1209, 0.1652] | 0.6323  [0.6307, 0.6340] |
| Whisper-large | 0.4960  [0.4663, 0.5253] | 0.2045  [0.1722, 0.2386] | 0.2540  [0.2235, 0.2847] | 0.6389  [0.6365, 0.6411] |
| RoBERTa | 0.6013  [0.5954, 0.6071] | 0.5225  [0.5013, 0.5435] | 0.5511  [0.5383, 0.5627] | 0.7000  [0.6972, 0.7027] |
| GPT embedding | **0.6333**  **[0.6292, 0.6377]** | 0.5628  [0.5460, 0.5792] | 0.5911  [0.5819, 0.6000] | **0.7228**  **[0.7210, 0.7245]** |
| Attention-based Fusion | 0.6034  [0.6005, 0.6062] | **0.6546**  **[0.6441, 0.6656]** | **0.6265**  **[0.6219, 0.6311]** | 0.7192  [0.7174, 0.7211] |
|  | **High risk vs. Non-high risk** | | | |
| Wav2vec | 0.6676  [0.6615, 0.6740] | 0.6329  [0.6099, 0.6546] | 0.6412  [0.6274, 0.6524] | 0.6566  [0.6528, 0.6599] |
| HuBERT | 0.6427  [0.6253, 0.6555] | 0.5839  [0.5483, 0.6172] | 0.5931  [0.5673, 0.6156] | 0.6294  [0.6221, 0.6362] |
| Whisper-chinese | 0.6890  [0.6810, 0.6969] | 0.7022  [0.6802, 0.7237] | 0.6874  [0.6784, 0.6959] | 0.6885  [0.6846, 0.6924] |
| Whisper-small | 0.7080  [0.7004, 0.7154] | 0.6742  [0.6483, 0.7002] | 0.6802  [0.6675, 0.6920] | 0.6942  [0.6893, 0.6988] |
| Whisper-medium | 0.6508  [0.6424, 0.6589] | 0.6049  [0.5731, 0.6376] | 0.6096  [0.5937, 0.6249] | 0.6328  [0.6283, 0.6370] |
| Whisper-large | 0.6730  [0.6644, 0.6815] | 0.6469  [0.6190, 0.6744] | 0.6458  [0.6322, 0.6583] | 0.6598  [0.6550, 0.6643] |
| RoBERTa | 0.7276  [0.7212, 0.7339] | 0.7995  [0.7836, 0.8148] | 0.7580  [0.7519, 0.7632] | 0.7483  [0.7447, 0.7516] |
| GPT embedding | **0.7929**  **[0.7884, 0.7974]** | 0.8201  [0.8106, 0.8298] | **0.8048**  **[0.8018, 0.8078]** | **0.8029**  **[0.8010, 0.8047]** |
| Attention-based Fusion | 0.7764  [0.7727, 0.7799] | **0.8270**  **[0.8200, 0.8340]** | 0.8001  [0.7975, 0.8027] | 0.7949  [0.7926, 0.7970] |

S4 Table shows the performance of the multidimensional deep learning classification model using different pre-trained models. The results compare the classification performance of extracting embeddings from audio and transcribed text using different pre-trained models. We tested Wav2vec 2.0, HuBERT, Whisper (including Whisper-small-Chinese-base, Whisper-small, Whisper-medium and Whisper-large-v3), which are pre-trained models for processing audio. For transcribed text, we selected RoBERTa and GPT models to extract text embeddings. The table presents the statistical values (displayed as Mean [95% Confidence Interval]) of model evaluation indicators in the prospective test set after 100 iterations.
