# Supplementary material for: Automating multi-label crisis detection in psychological support hotlines with pre-trained models
Source: PLOS Digit Health. 2026 May 13;5(5):e0001383. doi: 10.1371/journal.pdig.0001383 (PMC13170875; doi:10.1371/journal.pdig.0001383)
Supplement: S5 Table — (DOCX) [file pdig.0001383.s014.docx]

**S5 Table.** Performance evaluation for the multidimensional prediction with auditory temporal modeling

| **Methods** | **Precision** | **Recall** | **F1-Score** | **Accuracy** |
| --- | --- | --- | --- | --- |
|  | **Mood status: Depression vs. Normal** | | | |
| Wav2vec | 0.5124  [0.5076, 0.5171] | 0.8431  [0.8289, 0.8570] | 0.6349  [0.6322, 0.6374] | 0.5834  [0.5769, 0.5896] |
| HuBERT | 0.5046  [0.4990, 0.5100] | **0.8500**  **[0.8356, 0.8642]** | 0.6307  [0.6271, 0.6343] | 0.5717  [0.5625, 0.5803] |
| Whisper-chinese | 0.5313  [0.5263, 0.5364] | 0.8435  [0.8287, 0.8576] | 0.6494  [0.6460, 0.6526] | 0.6089  [0.6029, 0.6148] |
| Whisper-small | **0.5361**  **[0.5306, 0.5418]** | 0.8370  [0.8217, 0.8520] | **0.6506**  **[0.6476, 0.6536]** | **0.6139**  **[0.6073, 0.6205]** |
| Whisper-medium | 0.5035  [0.4979, 0.5090] | 0.8495  [0.8332, 0.8655] | 0.6290  [0.6261, 0.6319] | 0.5693  [0.5611, 0.5773] |
| Whisper-large | 0.5098  [0.5049, 0.5146] | 0.8495  [0.8367, 0.8624] | 0.6349  [0.6328, 0.6369] | 0.5798  [0.5729, 0.5863] |
|  | **Suicidal ideation: Yes vs. No** | | | |
| Wav2vec | 0.6423  [0.6370, 0.6474] | 0.9062  [0.8956, 0.9165] | 0.7500  [0.7478, 0.7521] | 0.6593  [0.6546, 0.6636] |
| HuBERT | 0.6252  [0.6198, 0.6307] | 0.9068  [0.8947, 0.9185] | 0.7381  [0.7352, 0.7408] | 0.6371  [0.6314, 0.6426] |
| Whisper-chinese | **0.6512**  **[0.6461, 0.6562]** | 0.8980  [0.8875, 0.9081] | 0.7534  [0.7505, 0.7561] | 0.6685  [0.6639, 0.6731] |
| Whisper-small | 0.6529  [0.6474, 0.6583] | 0.8964  [0.8850, 0.9075] | **0.7536**  **[0.7511, 0.7560]** | **0.6695**  **[0.6650, 0.6740]** |
| Whisper-medium | 0.6118  [0.6063, 0.6174] | **0.9325**  **[0.9219, 0.9427]** | 0.7369  [0.7356, 0.7383] | 0.6241  [0.6187, 0.6296] |
| Whisper-large | 0.6349  [0.6302, 0.6396] | 0.9191  [0.9088, 0.9290] | 0.7495  [0.7474, 0.7514] | 0.6535  [0.6492, 0.6576] |
|  | **Suicidal plan: Yes vs. No** | | | |
| Wav2vec | 0.4606  [0.4559, 0.4655] | **0.6408**  **[0.6174, 0.6637]** | 0.5286  [0.5210, 0.5355] | 0.5939  [0.5873, 0.6003] |
| HuBERT | 0.4468  [0.4260, 0.4635] | 0.5364  [0.5026, 0.5686] | 0.4791  [0.4552, 0.4997] | 0.6063  [0.5982, 0.6131] |
| Whisper-chinese | 0.4811  [0.4756, 0.4867] | 0.6263  [0.6036, 0.6481] | **0.5373**  **[0.5288, 0.5452]** | 0.6168  [0.6111, 0.6223] |
| Whisper-small | **0.4870**  **[0.4810, 0.4932]** | 0.6117  [0.5868, 0.6358] | 0.5336  [0.5250, 0.5418] | **0.6211**  **[0.6147, 0.6274]** |
| Whisper-medium | 0.4640  [0.4444, 0.4797] | 0.4730  [0.4321, 0.5120] | 0.4449  [0.4166, 0.4706] | 0.6193  [0.6131, 0.6249] |
| Whisper-large | 0.4700  [0.4578, 0.4789] | 0.5943  [0.5699, 0.6174] | 0.5176  [0.5036, 0.5281] | 0.6104  [0.6043, 0.6163] |
|  | **High risk vs. Non-high risk** | | | |
| Wav2vec | 0.5925  [0.5871, 0.5978] | **0.8718**  **[0.8592, 0.8842]** | 0.7032  [0.7008, 0.7054] | 0.6344  [0.6292, 0.6394] |
| HuBERT | 0.5770  [0.5711, 0.5828] | 0.8626  [0.8492, 0.8761] | 0.6890  [0.6856, 0.6923] | 0.6129  [0.6053, 0.6199] |
| Whisper-chinese | 0.6084  [0.6032, 0.6137] | 0.8595  [0.8460, 0.8722] | **0.7101**  **[0.7068, 0.7132]** | 0.6518  [0.6472, 0.6564] |
| Whisper-small | **0.6110**  **[0.6047, 0.6173]** | 0.8550  [0.8415, 0.8682] | 0.7100  [0.7069, 0.7131] | **0.6532**  **[0.6473, 0.6589]** |
| Whisper-medium | 0.5769  [0.5710, 0.5827] | 0.8627  [0.8473, 0.8777] | 0.6883  [0.6850, 0.6914] | 0.6123  [0.6058, 0.6185] |
| Whisper-large | 0.5856  [0.5799, 0.5913] | 0.8651  [0.8535, 0.8766] | 0.6962  [0.6940, 0.6983] | 0.6246  [0.6185, 0.6303] |

S5 Table shows the performance of the multidimensional deep learning classification model after incorporating BiLSTM (Bidirectional Long Short-Term Memory) for temporal sequence modeling. We evaluated the temporal modeling performance of Wav2vec 2.0, HuBERT, and the Whisper series (including Whisper-small-Chinese-base, Whisper-small, Whisper-medium, and Whisper-large-v3). The table presents the statistical values (displayed as Mean [95% Confidence Interval]) of model evaluation indicators in the prospective test set after 100 iterations.
