# Supplementary material for: Automating multi-label crisis detection in psychological support hotlines with pre-trained models
Source: PLOS Digit Health. 2026 May 13;5(5):e0001383. doi: 10.1371/journal.pdig.0001383 (PMC13170875; doi:10.1371/journal.pdig.0001383)
Supplement: S6 Table — (DOCX) [file pdig.0001383.s015.docx]

**S6 Table.** Performance evaluation for the multidimensional prediction with prompt engineering

| **Methods** | **Precision** | **Recall** | **F1-Score** | **Accuracy** |
| --- | --- | --- | --- | --- |
|  | **Mood status: Depression vs. Normal** | | | |
| DeepSeek-R1 | 0.4538  [0.4519, 0.4558] | **0.9913**  **[0.9913, 0.9913]** | 0.6226  [0.6208, 0.6245] | 0.4831  [0.4790, 0.4872] |
| DeepSeek-V3 | 0.4406  [0.4318, 0.4454] | 0.9827  [0.9628, 0.9939] | 0.6084  [0.5963, 0.6148] | 0.4559  [0.4391, 0.4652] |
| DeepSeek-R1-Distill-Qwen-32B | 0.4544  [0.4529, 0.4559] | 0.9714  [0.9680, 0.9758] | 0.6192  [0.6182, 0.6203] | 0.4860  [0.4827, 0.4894] |
| DeepSeek-R1-Distill-Llama-70B | 0.4617  [0.4567, 0.4668] | **0.9913**  **[0.9827, 0.9974]** | 0.6300  [0.6236, 0.6357] | 0.4991  [0.4894, 0.5088] |
| GPT-4o | 0.4448  [0.4430, 0.4466] | 0.9896  [0.9879, 0.9913] | 0.6137  [0.6118, 0.6158] | 0.4641  [0.4603, 0.4678] |
| GPT-4-turbo | 0.4655  [0.4632, 0.4682] | 0.9749  [0.9740, 0.9766] | 0.6301  [0.6280, 0.6324] | 0.5076  [0.5032, 0.5128] |
| Fine-tuned GPT-3.5-turbo | **0.6515**  **[0.6461, 0.6569]** | 0.8641  [0.8554, 0.8727] | **0.7428**  **[0.7375, 0.7488]** | **0.7426**  **[0.7374, 0.7486]** |
|  | **Suicidal ideation: Yes vs. No** | | | |
| DeepSeek-R1 | 0.7159  [0.7122, 0.7193] | **0.9815**  **[0.9776, 0.9848]** | 0.8280  [0.8242, 0.8312] | 0.7698  [0.7646, 0.7743] |
| DeepSeek-V3 | 0.6975  [0.6328, 0.7318] | 0.9056 [0.8172, 0.9518] | 0.7881  [0.7134, 0.8263] | 0.7255  [0.6298, 0.7750] |
| DeepSeek-R1-Distill-Qwen-32B | 0.7019  [0.6989, 0.7053] | 0.9512  [0.9465, 0.9558] | 0.8077  [0.8052, 0.8094] | 0.7445  [0.7408, 0.7471] |
| DeepSeek-R1-Distill-Llama-70B | 0.7139  [0.7103, 0.7203] | 0.9683  [0.9564, 0.9809] | 0.8218  [0.8173, 0.8266] | 0.7631  [0.7575, 0.7702] |
| GPT-4o | 0.7214  [0.7188, 0.7238] | 0.9637  [0.9604, 0.9663] | 0.8251  [0.8234, 0.8268] | 0.7695  [0.7669, 0.7721] |
| GPT-4-turbo | 0.7412  [0.7382, 0.7439] | 0.9525  [0.9485, 0.9564] | **0.8336**  **[0.8326, 0.8349]** | 0.7855  [0.7836, 0.7873] |
| Fine-tuned GPT-3.5-turbo | **0.8133**  **[0.8043, 0.8240]** | 0.8356  [0.8317, 0.8396] | 0.8243  [0.8193, 0.8292] | **0.7989**  **[0.7926, 0.8060]** |
|  | **Suicidal plan: Yes vs. No** | | | |
| DeepSeek-R1 | 0.5923  [0.5856, 0.5983] | **0.9433**  **[0.9361, 0.9505]** | **0.7277**  **[0.7229, 0.7337]** | 0.7449  [0.7382, 0.7512] |
| DeepSeek-V3 | 0.5795  [0.4576, 0.6413] | 0.7474  [0.5897, 0.8299] | 0.6528  [0.5156, 0.7230] | 0.7128  [0.5993, 0.7706] |
| DeepSeek-R1-Distill-Qwen-32B | 0.6059  [0.5966, 0.6148] | 0.8320  [0.8196, 0.8464] | 0.7011  [0.6907, 0.7115] | 0.7438  [0.7348, 0.7527] |
| DeepSeek-R1-Distill-Llama-70B | 0.5905  [0.5797, 0.6003] | 0.9052  [0.8866, 0.9309] | 0.7146  [0.7018, 0.7281] | 0.7389  [0.7274, 0.7501] |
| GPT-4o | 0.6223  [0.6126, 0.6336] | 0.8371  [0.8227, 0.8515] | 0.7138  [0.7018, 0.7257] | 0.7575  [0.7475, 0.7676] |
| GPT-4-turbo | **0.6277**  **[0.6124, 0.6391]** | 0.8381  [0.8289, 0.8464] | 0.7177  [0.7078, 0.7264] | **0.7616**  **[0.7505, 0.7702]** |
| Fine-tuned GPT-3.5-turbo | 0.5502  [0.5435, 0.5581] | 0.8763  [0.8670, 0.8866] | 0.6760  [0.6701, 0.6844] | 0.6965  [0.6894, 0.7054] |
|  | **High risk vs. Non-high risk** | | | |
| DeepSeek-R1 | 0.7353  [0.7239, 0.7459] | **0.9378**  **[0.9341, 0.9416]** | 0.8243  [0.8156, 0.8321] | 0.8011  [0.7899, 0.8112] |
| DeepSeek-V3 | 0.7664  [0.6298, 0.8397] | 0.7498  [0.6150, 0.8232] | 0.7580  [0.6223, 0.8313] | 0.7620  [0.6287, 0.8339] |
| DeepSeek-R1-Distill-Qwen-32B | 0.7780  [0.7716, 0.7852] | 0.8135  [0.8082, 0.8195] | 0.7953  [0.7901, 0.8006] | 0.7918  [0.7862, 0.7974] |
| DeepSeek-R1-Distill-Llama-70B | 0.7389  [0.7320, 0.7481] | 0.8809  [0.8659, 0.8966] | 0.8035  [0.7958, 0.8107] | 0.7858  [0.7784, 0.7933] |
| GPT-4o | **0.8190**  **[0.8091, 0.8286]** | 0.8367  [0.8255, 0.8502] | **0.8277**  **[0.8197, 0.8359]** | **0.8268**  **[0.8186, 0.8346]** |
| GPT-4-turbo | 0.7383  [0.7253, 0.7518] | 0.8884  [0.8816, 0.8944] | 0.8063  [0.7982, 0.8150] | 0.7877  [0.7769, 0.7993] |
| Fine-tuned GPT-3.5-turbo | 0.7513  [0.7441, 0.7586] | 0.8622  [0.8524, 0.8719] | 0.8029  [0.7961, 0.8106] | 0.7896  [0.7821, 0.7978] |
