# Supplementary material for: Automating multi-label crisis detection in psychological support hotlines with pre-trained models
Source: PLOS Digit Health. 2026 May 13;5(5):e0001383. doi: 10.1371/journal.pdig.0001383 (PMC13170875; doi:10.1371/journal.pdig.0001383)
Supplement: S7 Table — (DOCX) [file pdig.0001383.s016.docx]

**S7 Table.** Performance evaluation of multidimensional prediction models under a prevalence-aware simulation

| **Methods** | **Precision** | **Recall** | **F1-Score** | **Balanced-Accuracy** |
| --- | --- | --- | --- | --- |
|  | **Mood status: Depression vs. Normal** | | | |
| Wav2vec | 0.0996  [0.0960, 0.1032] | 0.6245  [0.5898, 0.6574] | 0.1689  [0.1629, 0.1748] | 0.6752  [0.6636, 0.6862] |
| Wav2vec* | 0.0692  [0.0668, 0.0718] | 0.8665  [0.8533, 0.8787] | 0.1279  [0.1237, 0.1322] | 0.6504  [0.6403, 0.6605] |
| HuBERT | 0.0972  [0.0910, 0.1032] | 0.6014  [0.5500, 0.6511] | 0.1628  [0.1530, 0.1721] | 0.6698  [0.6544, 0.6849] |
| HuBERT* | 0.0686  [0.0661, 0.0710] | **0.9159**  **[0.8935, 0.9364]** | 0.1272  [0.1229, 0.1314] | 0.6569  [0.6443, 0.6689] |
| Whisper-chinese | 0.0992  [0.0948, 0.1038] | 0.6530  [0.6192, 0.6858] | 0.1695  [0.1627, 0.1765] | 0.6790  [0.6678, 0.6902] |
| Whisper-chinese* | 0.0716  [0.0693, 0.0741] | 0.8372  [0.8193, 0.8543] | 0.1316  [0.1276, 0.1357] | 0.6566  [0.6474, 0.6656] |
| Whisper-small | 0.1118  [0.1072, 0.1165] | 0.6209  [0.5888, 0.6533] | 0.1869  [0.1799, 0.1941] | 0.6893  [0.6777, 0.7008] |
| Whisper-small* | 0.0713  [0.0690, 0.0737] | 0.8112  [0.7883, 0.8328] | 0.1306  [0.1268, 0.1346] | 0.6507  [0.6417, 0.6597] |
| Whisper-medium | 0.1040  [0.0984, 0.1097] | 0.6645  [0.6267, 0.7015] | 0.1750  [0.1668, 0.1832] | 0.6806  [0.6684, 0.6923] |
| Whisper-medium* | 0.0656  [0.0632, 0.0681] | 0.8712  [0.8569, 0.8848] | 0.1217  [0.1175, 0.1260] | 0.6325  [0.6208, 0.6442] |
| Whisper-large | 0.1048  [0.0999, 0.1097] | 0.6561  [0.6191, 0.6917] | 0.1758  [0.1692, 0.1822] | 0.6840  [0.6736, 0.6943] |
| Whisper-large* | 0.0674  [0.0653, 0.0695] | 0.8761  [0.8570, 0.8946] | 0.1248  [0.1212, 0.1285] | 0.6459  [0.6371, 0.6549] |
| RoBERTa | **0.1147**  **[0.1101, 0.1194]** | 0.6826  [0.6621, 0.7021] | **0.1944**  **[0.1878, 0.2012]** | **0.7107**  **[0.7027, 0.7184]** |
| GPT embedding | 0.1046  [0.1003, 0.1089] | 0.4744  [0.4496, 0.4994] | 0.1705  [0.1635, 0.1774] | 0.6415  [0.6313, 0.6518] |
| Attention-based Fusion | 0.0918  [0.0881, 0.0957] | 0.4628  [0.4414, 0.4845] | 0.1528  [0.1466, 0.1590] | 0.6230  [0.6133, 0.6329] |
| DeepSeek-R1 | 0.0466  [0.0460, 0.0472] | 0.9173  [0.9149, 0.9193] | 0.0887  [0.0876, 0.0897] | 0.5149  [0.5132, 0.5163] |
| DeepSeek-V3 | 0.0450  [0.0444, 0.0455] | 0.9334  [0.9274, 0.9399] | 0.0858  [0.0848, 0.0868] | 0.4981  [0.4966, 0.4996] |
| DeepSeek-R1-Distill-Qwen-32B | 0.0472  [0.0466, 0.0478] | 0.9092  [0.9037, 0.9141] | 0.0898  [0.0886, 0.0909] | 0.5208  [0.5180, 0.5233] |
| DeepSeek-R1-Distill-Llama-70B | 0.0497  [0.0490, 0.0505] | **0.9487**  **[0.9400, 0.9574]** | 0.0945  [0.0931, 0.0960] | 0.5455  [0.5405, 0.5505] |
| GPT-4o | 0.0448  [0.0442, 0.0454] | 0.9173  [0.9149, 0.9193] | 0.0854  [0.0844, 0.0864] | 0.4961  [0.4946, 0.4975] |
| GPT-4-turbo | 0.0495  [0.0489, 0.0502] | 0.9100  [0.9047, 0.9147] | 0.0939  [0.0927, 0.0951] | 0.5418  [0.5388, 0.5446] |
| Fine-tuned GPT-3.5-turbo | **0.1055**  **[0.1027, 0.1083]** | 0.6795  [0.6633, 0.6963] | **0.1825**  **[0.1779, 0.1873]** | **0.7035**  **[0.6955, 0.7119]** |
|  | **Suicidal ideation: Yes vs. No** | | | |
| Wav2vec | 0.3004  [0.2945, 0.3065] | 0.7092  [0.6897, 0.7284] | 0.4174  [0.4132, 0.4215] | 0.6376  [0.6334, 0.6416] |
| Wav2vec* | 0.2582  [0.2538, 0.2626] | 0.8582  [0.8437, 0.8726] | 0.3953  [0.3908, 0.3996] | 0.6094  [0.6032, 0.6155] |
| HuBERT | 0.2701  [0.2627, 0.2777] | 0.7260  [0.6943, 0.7577] | 0.3841  [0.3782, 0.3899] | 0.5960  [0.5876, 0.6041] |
| HuBERT* | 0.2453  [0.2412, 0.2495] | 0.8718  [0.8546, 0.8886] | 0.3812  [0.3768, 0.3856] | 0.5880  [0.5808, 0.5951] |
| Whisper-chinese | 0.2947  [0.2888, 0.3006] | 0.6446  [0.6217, 0.6673] | 0.3987  [0.3937, 0.4036] | 0.6191  [0.6143, 0.6239] |
| Whisper-chinese* | 0.2522  [0.2485, 0.2560] | 0.8024  [0.7857, 0.8184] | 0.3822  [0.3780, 0.3863] | 0.5939  [0.5882, 0.5995] |
| Whisper-small | 0.2993  [0.2933, 0.3052] | 0.6215  [0.5978, 0.6448] | 0.3980  [0.3922, 0.4034] | 0.6194  [0.6146, 0.6240] |
| Whisper-small* | 0.2535  [0.2499, 0.2573] | 0.8011  [0.7824, 0.8201] | 0.3831  [0.3794, 0.3868] | 0.5955  [0.5904, 0.6005] |
| Whisper-medium | 0.2748  [0.2685, 0.2811] | 0.7416  [0.7099, 0.7719] | 0.3915  [0.3860, 0.3963] | 0.6081  [0.6023, 0.6136] |
| Whisper-medium* | 0.2348  [0.2308, 0.2390] | **0.9004**  **[0.8860, 0.9142]** | 0.3708  [0.3670, 0.3747] | 0.5691  [0.5624, 0.5758] |
| Whisper-large | 0.2852  [0.2803, 0.2903] | 0.7151  [0.6845, 0.7453] | 0.3998  [0.3952, 0.4042] | 0.6208  [0.6165, 0.6249] |
| Whisper-large* | 0.2547  [0.2511, 0.2584] | 0.8971  [0.8815, 0.9122] | 0.3953  [0.3915, 0.3991] | 0.6096  [0.6041, 0.6151] |
| RoBERTa | 0.3427  [0.3362, 0.3496] | 0.7422  [0.7236, 0.7598] | 0.4643  [0.4593, 0.4693] | 0.6841  [0.6798, 0.6883] |
| GPT embedding | **0.4070**  **[0.4015, 0.4126]** | 0.6577  [0.6464, 0.6696] | **0.5006**  **[0.4966, 0.5045]** | **0.7047**  **[0.7014, 0.7080]** |
| Attention-based Fusion | 0.3903  [0.3854, 0.3953] | 0.6626  [0.6500, 0.6751] | 0.4894  [0.4846, 0.4943] | 0.6977  [0.6935, 0.7020] |
| DeepSeek-R1 | 0.3386  [0.3369, 0.3404] | **0.9557**  **[0.9518, 0.9596]** | 0.5001  [0.4977, 0.5025] | 0.7391  [0.7364, 0.7418] |
| DeepSeek-V3 | 0.3191  [0.3079, 0.3295] | 0.8574  [0.8444, 0.8694] | 0.4640  [0.4499, 0.4772] | 0.6867  [0.6692, 0.7032] |
| DeepSeek-R1-Distill-Qwen-32B | 0.3217  [0.3200, 0.3234] | 0.9238  [0.9212, 0.9264] | 0.4772  [0.4750, 0.4794] | 0.7126  [0.7104, 0.7150] |
| DeepSeek-R1-Distill-Llama-70B | 0.3352  [0.3336, 0.3369] | 0.9441  [0.9378, 0.9503] | 0.4946  [0.4924, 0.4968] | 0.7324  [0.7297, 0.7350] |
| GPT-4o | 0.3393  [0.3383, 0.3404] | 0.9219  [0.9183, 0.9254] | 0.4961  [0.4946, 0.4975] | 0.7314  [0.7298, 0.7330] |
| GPT-4-turbo | 0.3601  [0.3593, 0.3609] | 0.8891  [0.8850, 0.8933] | 0.5125  [0.5113, 0.5138] | **0.7425**  **[0.7411, 0.7439]** |
| Fine-tuned GPT-3.5-turbo | **0.4350**  **[0.4305, 0.4395]** | 0.6454  [0.6412, 0.6495] | **0.5194**  **[0.5153, 0.5234]** | 0.7151  [0.7122, 0.7179] |
|  | **Suicidal plan: Yes vs. No** | | | |
| Wav2vec | 0.0320  [0.0255, 0.0389] | 0.1737  [0.1425, 0.2053] | 0.0522  [0.0421, 0.0626] | 0.5388  [0.5245, 0.5534] |
| Wav2vec* | 0.0290  [0.0265, 0.0317] | 0.5947  [0.5408, 0.6482] | 0.0550  [0.0502, 0.0600] | 0.6136  [0.5918, 0.6348] |
| HuBERT | 0.0359  [0.0269, 0.0456] | 0.2075  [0.1608, 0.2558] | 0.0583  [0.0444, 0.0732] | 0.5705  [0.5517, 0.5900] |
| HuBERT* | 0.0236  [0.0204, 0.0267] | 0.4350  [0.3777, 0.4913] | 0.0445  [0.0386, 0.0504] | 0.5634  [0.5399, 0.5862] |
| Whisper-chinese | 0.0423  [0.0365, 0.0487] | 0.3403  [0.2918, 0.3908] | 0.0725  [0.0631, 0.0826] | 0.6007  [0.5802, 0.6215] |
| Whisper-chinese* | 0.0376  [0.0347, 0.0406] | 0.6513  [0.6075, 0.6940] | 0.0705  [0.0652, 0.0759] | 0.6629  [0.6417, 0.6829] |
| Whisper-small | 0.0444  [0.0369, 0.0523] | 0.2778  [0.2338, 0.3225] | 0.0730  [0.0617, 0.0846] | 0.5861  [0.5676, 0.6046] |
| Whisper-small* | 0.0408  [0.0374, 0.0443] | **0.6617**  **[0.6112, 0.7115]** | 0.0760  [0.0699, 0.0823] | **0.6793**  **[0.6580, 0.7007]** |
| Whisper-medium | 0.0169  [0.0104, 0.0244] | 0.0683  [0.0433, 0.0973] | 0.0257  [0.0162, 0.0367] | 0.5107  [0.5000, 0.5229] |
| Whisper-medium* | 0.0385  [0.0340, 0.0433] | 0.5237  [0.4653, 0.5810] | 0.0692  [0.0618, 0.0763] | 0.6397  [0.6183, 0.6605] |
| Whisper-large | 0.0231  [0.0165, 0.0300] | 0.1185  [0.0865, 0.1527] | 0.0367  [0.0266, 0.0475] | 0.5205  [0.5069, 0.5348] |
| Whisper-large* | 0.0291  [0.0262, 0.0319] | 0.4983  [0.4572, 0.5388] | 0.0546  [0.0493, 0.0598] | 0.5896  [0.5714, 0.6076] |
| RoBERTa | 0.0474  [0.0405, 0.0543] | 0.3048  [0.2640, 0.3457] | 0.0802  [0.0691, 0.0914] | 0.5936  [0.5744, 0.6128] |
| GPT embedding | **0.0723**  **[0.0638, 0.0811]** | 0.3610  [0.3233, 0.3997] | 0.1190  [0.1057, 0.1331] | 0.6387  [0.6204, 0.6573] |
| Attention-based Fusion | 0.0648  [0.0585, 0.0713] | 0.4800  [0.4352, 0.5238] | **0.1135**  **[0.1026, 0.1244]** | 0.6762  [0.6540, 0.6978] |
| DeepSeek-R1 | 0.0658  [0.0628, 0.0689] | 0.9258  [0.9022, 0.9480] | 0.1226  [0.1171, 0.1282] | **0.8448**  **[0.8326, 0.8564]** |
| DeepSeek-V3 | 0.0706  [0.0630, 0.0781] | 0.6378  [0.5878, 0.6853] | 0.1261  [0.1130, 0.1392] | 0.7148  [0.6780, 0.7487] |
| DeepSeek-R1-Distill-Qwen-32B | 0.0592  [0.0558, 0.0626] | 0.6832  [0.6580, 0.7070] | 0.1086  [0.1025, 0.1147] | 0.7431  [0.7303, 0.7553] |
| DeepSeek-R1-Distill-Llama-70B | 0.0549  [0.0518, 0.0580] | 0.7657  [0.7352, 0.7952] | 0.1022  [0.0966, 0.1078] | 0.7642  [0.7492, 0.7786] |
| GPT-4o | **0.0715**  **[0.0661, 0.0769]** | 0.6910  [0.6475, 0.7333] | **0.1291**  **[0.1196, 0.1386]** | 0.7640  [0.7419, 0.7856] |
| GPT-4-turbo | 0.0701  [0.0657, 0.0746] | 0.6780  [0.6505, 0.7047] | 0.1265  [0.1189, 0.1343] | 0.7567  [0.7427, 0.7704] |
| Fine-tuned GPT-3.5-turbo | 0.0562  [0.0538, 0.0587] | **0.9480**  **[0.9270, 0.9672]** | 0.1059  [0.1015, 0.1104] | 0.8314  [0.8209, 0.8410] |
|  | **High risk vs. Non-high risk** | | | |
| Wav2vec | 0.0284  [0.0262, 0.0308] | 0.6100  [0.5625, 0.6600] | 0.0537  [0.0498, 0.0578] | 0.6450  [0.6238, 0.6664] |
| Wav2vec* | 0.0222  [0.0212, 0.0232] | **0.8975**  **[0.8675, 0.9275]** | 0.0432  [0.0414, 0.0451] | 0.6486  [0.6329, 0.6641] |
| HuBERT | 0.0273  [0.0244, 0.0303] | 0.5725  [0.5175, 0.6275] | 0.0511  [0.0462, 0.0562] | 0.6235  [0.6003, 0.6465] |
| HuBERT* | 0.0204  [0.0194, 0.0213] | 0.8750  [0.8375, 0.9100] | 0.0398  [0.0380, 0.0416] | 0.6204  [0.6029, 0.6377] |
| Whisper-chinese | 0.0329  [0.0304, 0.0355] | 0.7050  [0.6575, 0.7550] | 0.0625  [0.0579, 0.0673] | 0.6900  [0.6684, 0.7115] |
| Whisper-chinese* | 0.0229  [0.0217, 0.0240] | 0.8600  [0.8200, 0.8950] | 0.0445  [0.0424, 0.0467] | 0.6532  [0.6349, 0.6705] |
| Whisper-small | 0.0364  [0.0331, 0.0398] | 0.6850  [0.6325, 0.7350] | 0.0686  [0.0625, 0.0749] | 0.6995  [0.6755, 0.7235] |
| Whisper-small* | 0.0231  [0.0220, 0.0243] | 0.8550  [0.8175, 0.8925] | 0.0450  [0.0428, 0.0473] | 0.6543  [0.6365, 0.6721] |
| Whisper-medium | 0.0278  [0.0250, 0.0309] | 0.6050  [0.5525, 0.6575] | 0.0527  [0.0474, 0.0582] | 0.6327  [0.6104, 0.6548] |
| Whisper-medium* | 0.0204  [0.0195, 0.0213] | 0.8725  [0.8375, 0.9050] | 0.0398  [0.0380, 0.0416] | 0.6186  [0.6023, 0.6345] |
| Whisper-large | 0.0306  [0.0279, 0.0333] | 0.6650  [0.6100, 0.7200] | 0.0580  [0.0531, 0.0629] | 0.6688  [0.6463, 0.6905] |
| Whisper-large* | 0.0202  [0.0191, 0.0213] | 0.8350  [0.7950, 0.8725] | 0.0393  [0.0372, 0.0415] | 0.6108  [0.5916, 0.6303] |
| RoBERTa | 0.0392  [0.0369, 0.0414] | 0.8025  [0.7625, 0.8425] | 0.0744  [0.0704, 0.0786] | 0.7501  [0.7315, 0.7680] |
| GPT embedding | **0.0527**  **[0.0494, 0.0561]** | 0.7875  [0.7450, 0.8276] | **0.0986**  **[0.0926, 0.1047]** | 0.7867  [0.7659, 0.8070] |
| Attention-based Fusion | 0.0507  [0.0482, 0.0530] | 0.8400  [0.8050, 0.8750] | 0.0955  [0.0910, 0.0998] | **0.8016**  **[0.7832, 0.8187]** |
| DeepSeek-R1 | 0.0393  [0.0381, 0.0405] | **0.9200**  **[0.8925, 0.9450]** | 0.0754  [0.0731, 0.0776] | 0.7930  [0.7793, 0.8061] |
| DeepSeek-V3 | 0.0577  [0.0524, 0.0627] | 0.7500  [0.7000, 0.7975] | 0.1065  [0.0969, 0.1157] | 0.7620  [0.7306, 0.7913] |
| DeepSeek-R1-Distill-Qwen-32B | 0.0488  [0.0464, 0.0512] | 0.7975  [0.7550, 0.8375] | 0.0920  [0.0874, 0.0965] | 0.7839  [0.7634, 0.8039] |
| DeepSeek-R1-Distill-Llama-70B | 0.0399  [0.0382, 0.0416] | 0.8650  [0.8275, 0.9000] | 0.0764  [0.0731, 0.0794] | 0.7784  [0.7599, 0.7959] |
| GPT-4o | **0.0636**  **[0.0608, 0.0662]** | 0.8375  [0.8000, 0.8725] | **0.1181**  **[0.1129, 0.1230]** | **0.8273**  **[0.8088, 0.8448]** |
| GPT-4-turbo | 0.0399  [0.0381, 0.0415] | 0.8700  [0.8325, 0.9050] | 0.0762  [0.0728, 0.0794] | 0.7791  [0.7603, 0.7967] |
| Fine-tuned GPT-3.5-turbo | 0.0444  [0.0425, 0.0460] | 0.8850  [0.8475, 0.9175] | 0.0845  [0.0810, 0.0876] | 0.8014  [0.7831, 0.8179] |

To reflect the natural imbalance of a psychological support hotline, we conducted a prevalence-aware simulation based on the 2023 Hangzhou Hotline statistics (276 high-risk calls out of 18,245 total calls, representing a ~1.5% prevalence). The evaluation was performed by randomly sampling from the prospective test set to achieve a ratio of 4 high-risk calls to 270 controls. The blue shading areas represent Deep Learning models developed using extracted embeddings from audio and transcribed text. Models marked with an asterisk indicate the implementation of a BiLSTM (Bidirectional Long Short-Term Memory) architecture to capture sequential dependencies in audio embeddings, rather than using simple mean-pooling. The green shading areas represent Large Language Models (LLMs) evaluated using the prompt engineering. Values are displayed as Mean [95% Confidence Interval] derived from the aggregated results of 100 simulated predictions. For methods using LLM with prompt engineering, to ensure statistical consistency, we performed 20 random samplings across each of the 5 original evaluation iterations, yielding a total of 100 simulated data points.
