# Supplementary material for: Automating multi-label crisis detection in psychological support hotlines with pre-trained models
Source: PLOS Digit Health. 2026 May 13;5(5):e0001383. doi: 10.1371/journal.pdig.0001383 (PMC13170875; doi:10.1371/journal.pdig.0001383)
Supplement: Attachment — Submitted filename: Response_to_Reviewers.docx [file pdig.0001383.s018.docx]

**Response to Editor and Reviewers**

We sincerely thank editor and reviewers for their thorough evaluation and constructive feedback on our manuscript. The insightful comments have been invaluable in improving the quality and clarity of our work. To address these comments, we have conducted supplementary experiments, included more methodology details and strengthened the discussion. We have undertaken a substantial revision to address all comments and believe the manuscript has been substantially improved and strengthened. All modifications are highlighted with yellow background in the revised manuscript. In this point-by-point response, reviewer comments are presented in black font, and our responses are in blue font.

Additional Editor Comments:

This manuscript evaluates automated multi-label crisis detection on 1,057 psychological hotline calls (2022–2023), comparing embedding-based deep learning models (audio/text pretrained representations) with LLM prompt-based prediction, and includes an expert review of generated explanations. However, the following concerns should be addressed before publication:

1.Unclear dataset generation and questionnaire administration. The manuscript defines and computes depression, high-risk status, and risk-factor scores, but does not specify how the 20-item questionnaire was administered in real hotline calls (routine scripted questions vs operator judgment vs post-call coding). This ambiguity directly affects label validity and data quality.

**Response:**

We appreciate editor’s insightful comment regarding the administration of the 20-item questionnaire. We recognize that the validity of our labels depends on the rigor of the data collection process in a high-pressure hotline environment.

The questionnaire was administered using a semi-structured clinical interview approach rather than a rigid script. This ensured therapeutic rapport was maintained while systematically capturing essential clinical data. The process followed these steps:

1. **Professional Administration:** All calls were handled by operators who had undergone specialized suicide risk assessment training.
2. **Guided Exploration:** During the consultation, operators used the 20 items in S1 Table as a framework, probing deeper into relevant clinical dimensions (e.g., mood, hopelessness, or intent) as they naturally surfaced during the conversation.
3. **Post-call Labeling:** Immediately following the conclusion of each call, operators completed standardized electronic logs and assigned the four study labels (mood status, suicidal ideation, suicidal plan, and high-risk status) according to the explicit criteria listed in S1 Table. This ensures that the labels reflect the totality of the professional interaction and provide a reliable ground truth.

We have clarified this process in the section of “Data collection and selection” on Page 15 to ensure transparency regarding the data's clinical validity:

“The Depression and Suicide Risk assessment questionnaire (S1 Table) was administered via a semi-structured approach. To maintain working alliance, operators did not read the questionnaire as a rigid script. Instead, they used the items as a systematic framework to guide the conversation, probing for specific risk factors as they naturally emerged. Following each call, operators utilized their clinical judgment and the caller's disclosures to complete a standardized assessment log, which served as the basis for the labels used in this study.”

2.Label-definition mismatch between questionnaire and LLM prompts. “High-risk” is operationalized in the dataset via explicit logical criteria involving suicidal ideation, suicidal plan, and a risk-factor, while the prompt redefines “high-risk” as a broader “immediate and severe crisis” concept with a 7-level ladder later collapsed to binary.

**Response:**

We thank editor for this meticulous observation. We acknowledge the difference in phrasing between the questionnaire criteria and the LLM prompt instructions. However, we would like to clarify that these two approaches are conceptually and operationally aligned for the following reasons:

1. **7-Level Ladder as a Chain-of-Thought (CoT) Mechanism:** The 7-level risk hierarchy in the prompt was designed as a CoT strategy. LLMs perform significantly better on complex clinical tasks when they are guided to reason through intermediate steps. By asking the model to first identify where a caller sits on a 7-level ladder—ranging from “no thoughts” to “immediate intent/action”—we force the model to explicitly analyze the same core components used in the dataset's operational definition: suicidal ideation, plans, and immediate behavior.
2. **Mapping to Clinical Criteria:** Although the prompt uses broader language like “immediate and severe crisis”, the internal logic directly mirrors the high-risk criteria used by the hotline. For example, levels 3 through 7 on our prompt’s ladder explicitly require the presence of a “plan” or “intent”, which directly corresponds to the primary high-risk criterion in our dataset (Ideation + Plan).
3. **Binary Simplification for Clinical Utility:** The decision to collapse the model’s output into a binary “High-risk vs. Non-high-risk” classification was intentional. In a real-world hotline setting, the most critical task for an AI “co-pilot” is to provide a clear, actionable flag to help operators prioritize urgent cases and allocate life-saving resources. Furthermore, this binary output was necessary to benchmark the LLM's performance against our deep learning models and the ground-truth labels recorded by operators.

In summary, the 7-level ladder serves as a computational scaffold to help the LLM navigate the complex, non-linear reasoning required to satisfy the hotline's formal “high-risk” definition.

We have now included the following text in the section of “LLM-based prediction via prompt engineering” on Page 18 to explain this reasoning:

“To enhance inferential rigor, we utilized CoT prompting [30] by incorporating a 7-level risk hierarchy as a reasoning scaffold. This hierarchy decomposes the “high-risk” logical criteria—which involve ideation, planning, and specific risk scores—into a progressive sequence of clinical indicators. This approach assists LLMs in performing explicit reasoning over the same clinical dimensions used in manual labeling while ultimately producing a binary output suitable for rapid crisis prioritization. The full prompt configurations are provided in S2 Table.”

3.Transcript creation and “caller-only” extraction are not reproducible. Transcripts are produced by a third-party ASR system, and the analysis uses caller-only text, but the manuscript does not document speaker separation (diarization/role tagging/heuristics), error rates, or any ASR quality control.

**Response:**

We appreciate the editor’s request for technical clarification regarding our transcription and speaker extraction process. We have updated the manuscript to provide the following details to ensure reproducibility:

1. **Deterministic Speaker Separation:** The hotline audio was recorded in a dual-channel (stereo) format, where the left channel exclusively captured the caller’s audio and the right channel captured the operator’s audio. Consequently, “caller-only” extraction was achieved by isolating the left channel prior to processing. This approach bypasses the need for complex speaker diarization or heuristic role tagging, ensuring 100% accuracy in speaker attribution.

In the section of “Data collection and selection” on Page 15, we have included the following text:

“The recordings were captured in a dual-channel format, with the caller and the operator recorded on independent tracks. By specifically extracting the caller's channel (Left), we achieved deterministic speaker isolation. The caller’s audio was then preprocessed through removing silence periods, denoising, and loudness normalization.”

2. **ASR Tool Selection:** Given the massive scale of our dataset—totaling 260.54 hours of audio (126.83 hours in 2022 and 133.71 hours in 2023) and over 4 million Chinese characters (1,862,083 in 2022; 2,138,958 in 2023)—manual correction was not feasible. To ensure high-quality transcription, we conducted a pilot comparison of three leading Chinese ASR services (iFlytek, Baidu AI Cloud, and Aliyun). iFlytek was selected based on its superior performance in handling the specific acoustic conditions and terminologies of the hotline environment.
3. **ASR Quality and Model Robustness:** While we did not perform exhaustive post-transcription manual error correction, recent literature and our own observations suggest that Large Language Models (LLMs) are inherently robust to minor ASR errors (such as phonetic substitutions or typos) due to their deep contextual understanding (*Hu Y, Chen C, Yang C-HH, Li R, Zhang C, Chen P-Y, et al. Large Language Models are Efficient Learners of Noise-Robust Speech Recognition, International Conference on Learning Representations, ICLR, 2024; Shapira O, Chazan S, Cohen ADN. Measuring the Effect of Transcription Noise on Downstream Language Understanding Tasks. Proceedings of the 63rd Annual Meeting of the Association for Computational Linguistics (Volume 1: Long Papers), 2025, p. 29978–30004*).

We acknowledge that the lack of manual ASR quality control is a limitation. We have included the following text in the Discussion section on Page 14:

“Fourth, while we utilized a high-performance ASR system, the transcripts did not undergo manual correction. Although LLMs are robust to ASR noise, future work involving fine-grained linguistic analysis may benefit from human-in-the-loop correction.”

4.Statistical claims are not supported. The paper reports averages over repeated runs, but variance/CI reporting is inconsistent and “no significant gain” statements for fusion are not backed by clear tests, effect sizes, or confidence intervals. The supplement’s use of independent-samples t-tests across repeated runs is not appropriate for comparing methods on the same fixed test set, and multiple comparisons across many models/labels are not controlled, so p<0.05 claims are not reliable.

**Response:**

We sincerely thank the editor for this critical evaluation of our statistical methodology. We would like to clarify that our repeated iterations (100 for deep learning, 5 for LLMs) were designed to evaluate model stability against stochasticity in initialization, training dynamics, and LLM output generation, rather than data sampling variability. Since there is no inherent one-to-one correspondence between the $i$-th run of different architectures, we treat these as independent samples for performance comparison.

However, we recognize that our initial reporting lacked sufficient detail regarding variance and multi-comparison controls. To address this, we have upgraded our statistical framework in the revised manuscript:

1. **Robust Estimation with Bootstrap Confidence Intervals:** Instead of relying on standard deviation, we now report 95% Confidence Intervals (95% CIs) for all primary performance metrics (Precision, Recall, F1-Score, and Accuracy). These CIs were computed using the Bootstrap method (10,000 resamples with replacement). This approach provides a more stable estimate of model performance and allows for a clearer visualization of the variance across different training iterations.

The updated performance metrics for the deep learning classification frameworks and the prompt engineering strategies are detailed in S4 Table and S6 Table of the Supplementary Material, respectively, with results now displayed as Mean [95% Confidence Interval].

2. **Robust Statistical Comparisons:** To accommodate the disparate sample sizes and variances across models, we have replaced standard $t$-tests with the following: Welch’s $t$-test for large-scale (100 vs. 100) comparisons, Permutation tests for mixed-scale (100 vs. 5) comparisons, and Wilcoxon Rank Sum tests for small-scale (5 vs. 5) comparisons.
3. **Control for Multiple Comparisons:** To mitigate the risk of Type I errors (false positives) arising from multiple comparisons across various models and labels, we have implemented the Holm-Bonferroni correction. All reported $p$-values have been adjusted within each label category. A result is only claimed as “statistically significant” if its adjusted $p$-value remains below the $\alpha=0.05$ threshold.

The results of above statistical comparison framework are updated in S4–S7 Figs of the Supplementary Material.

We have included the following text in the “Model evaluation” section on Page 20:

“The overall performance indicators were obtained by aggregating results across all 100 iterations (for deep learning models) and 5 iterations (for LLMs), with results presented as means and 95% Confidence Intervals derived via the Bootstrap method. Comprehensive performance matrices are provided in S4 Table and S6 Table. Furthermore, we implemented a rigorous statistical comparison framework: Welch’s t-test was used for large-scale (100 vs. 100) model comparisons; Permutation tests were employed for mixed-scale (100 vs. 5) comparisons; and the Wilcoxon Rank Sum test was applied for small-scale (5 vs. 5) comparisons. To mitigate the risk of Type I errors from multiple comparisons, all p-values were adjusted using the Holm-Bonferroni correction. Comprehensive statistical results are detailed in S4–S7 Figs.”

5.Case-control sampling limits external validity. The dataset is artificially balanced (roughly half high-risk via matched controls), while real-world hotline prevalence is likely much lower. Precision/recall, thresholds, and operational utility derived from this setting will not transfer without prevalence-aware evaluation.

**Response:**

We thank the editor for this critical observation regarding the external validity of our findings. We acknowledge that our initial balanced dataset was designed to maximize the model's ability to learn high-risk features, but it does not reflect the natural prevalence in a real-world hotline setting.

To address this concern and evaluate the model’s operational utility, we conducted a prevalence-aware simulation based on actual 2023 hotline statistics. In 2023, the Hangzhou Hotline received a total of 18,245 calls, of which 276 were clinically identified as high-risk (a prevalence of approximately 1.5%). To simulate this imbalance, we re-evaluated our best-performing model (the GPT-embedding neural network) by randomly sampling calls from our prospective test set to reflect a ratio of 4 high-risk calls to 270 controls.

As illustrated in the supplementary performance table (S7 Table), taking the prevalence-aware evaluation results of GPT embedding-based deep learning models as an example—representing the peak performance among all embedding-based neural network frameworks:

1. **Impact on Precision and F1-Score:** As expected in a low-prevalence scenario, the Precision and F1-score decreased across all four tasks. This is a common mathematical artifact when the “true positive” pool is extremely small compared to the “true negative” pool.
2. **Robustness of Recall and Accuracy:** For “High Risk vs. Non-High Risk” classification task, the model maintained a Recall of 78.75% [74.50%, 82.76%] and a Balanced-Accuracy of 78.67% [76.59%, 80.70%].
3. **Clinical Utility:** In the context of psychological crisis intervention, prioritizing Recall is paramount to ensure that high-risk individuals are not missed (minimizing Type II errors). A recall of nearly 79% in a simulated real-world environment suggests that the model can effectively serve as a “safety net” or screening tool to alert human operators to potential crises that require immediate attention.

The comprehensive performance metrics for all evaluated models under these simulated real-world conditions are presented in the added S7 Table of the Supplementary Material.

We have included the following text in the “Multidimensional prediction with crafted models” section on Page 8:

“To evaluate model’s generalizability to real-world settings where high-risk calls are infrequent, we performed a prevalence-aware simulation. We simulated a 1.5% high-risk prevalence—mirroring 2023 hotline statistics—with a 4:270 high-risk-to-control ratio. Comprehensive performance metrics for each method under these imbalanced conditions are provided in S7 Table. We observed that while precision naturally declined due to low prevalence, GPT embedding still maintained high recall (78.75% [74.50%, 82.76%]) and accuracy (78.67% [76.59%, 80.70%]) in the High Risk vs. Non-High Risk classification task. This suggests the model remains an effective decision-support tool for prioritizing urgent interventions even in imbalanced conditions.”

We have included the following text in the Discussion section on Page 13:

“While our study utilized a matched case-control design to optimize feature learning, this may not fully reflect model performance in real-world settings where high-risk callers represent a small minority (approximately 1.5%). Nevertheless, our prevalence-aware simulation demonstrates that the GPT-embedding deep learning model remains a robust screening instrument. Although its Positive Predictive Value (PPV) is sensitive to such skewed distributions, the model maintains a high recall. In the high-stakes context of psychological crisis intervention, prioritizing recall is paramount to ensuring that no high-risk individual is overlooked. The high sensitivity of this framework suggests its potential to function as an automated “safety net”, effectively alerting operators to latent crises that necessitate immediate clinical attention.”

We have included the following text in the Discussion section on Page 14:

“Third, while our prevalence-aware simulation validated the GPT-embedding deep learning model as a high-recall screening tool, its PPV remains sensitive to the low-prevalence typical of real-world settings. Future deployment will necessitate cost-sensitive learning or advanced data augmentation to manage extreme class imbalances without compromising clinical utility.”

6.Audio baseline likely handicaps acoustic information. The audio pipeline averages embeddings over 10-second segments, collapsing temporal dynamics that plausibly encode distress escalation and hesitation patterns. Concluding that “acoustic features add negligible value” is not justified without stronger temporal audio modeling or ablations that isolate where acoustic signal is lost.

**Response:**

We sincerely appreciate the editor's insightful suggestion regarding the potential loss of temporal information in our original audio processing pipeline. We agree that simple averaging of 10-second segments may mask critical paralinguistic cues such as hesitation patterns or distress escalation.

To address this, we conducted a supplementary comparative analysis by implementing a BiLSTM (Bidirectional Long Short-Term Memory) architecture. This model variant was designed specifically to preserve and model the sequential dependencies within the audio pre-trained model-derived embeddings, rather than treating them as static averages.

As illustrated in the supplementary performance table (S5 Table), taking the results of temporal modeling based on Whisper-small-derived embeddings as an example—which emerged as the superior audio pre-trained model within our neural network framework:

1. **Significant Recall Gains:** The BiLSTM model achieved a substantial increase in Recall across all labels. For instance, the Recall for the “High Risk vs. Non-High Risk” classification improved from 67.42% [64.83%, 70.02%] to 85.50% [84.15%, 86.82%].
2. **F1-Score and Reliability:** We observed a clear improvement in F1-scores, particularly in identifying "Suicidal plans," which rose from 39.27% [36.99%, 41.51%] to 53.36% [52.50%, 54.18%]. This confirms that temporal modeling indeed captures a stronger acoustic signal than simple pooling.
3. **Modality Comparison:** However, even with this enhanced temporal modeling, the audio-only performance remained significantly lower than the text-only GPT-embedding framework. For the “High Risk vs. Non-High Risk” classification task, the text-based model’s F1-score (80.48% [80.18%, 80.78%]) still substantially outperforms the best audio-only result (71.00% [70.69%, 71.31%]).

These additional experiments demonstrate that while acoustic temporal dynamics provide valuable information, the semantic content encoded in the text remains the most robust and predictive signal for crisis detection in this hotline context.

The comprehensive performance metrics for auditory temporal modeling across various pre-trained models are presented in the added S5 Table of the Supplementary Material.

We have included the following text in the “Multidimensional prediction with crafted models” section on Page 8:

“By adopting Bidirectional Long Short-Term Memory (BiLSTM) strategies to preserve temporal dynamics, we observed a significant improvement in recall for auditory models; however, the corresponding increase in F1-score remained modest (see S5 Table).”

We have included the following text in the Discussion section on Page 12:

“While adopting BiLSTM strategies to preserve time-series information significantly improved acoustic-based recall, the overall performance ceiling remained firmly dictated by the text modality.”

We have included the following text in the Discussion section on Page 14:

“Additionally, audio-text fusion experiments did not yield statistically significant performance improvements. Future work should move beyond modality-specific temporal modeling toward fine-grained, cross-modal attention mechanisms. Such strategies could enable the model to dynamically align transient acoustic markers with specific high-risk linguistic segments, thereby enhancing the synergy between vocal urgency and articulated intent.”

We have included the following text in the “Deep learning prediction framework with pre-trained model” section on Page 16:

“First, an averaging operation was applied to these embeddings to form a global representation of the call. However, to better capture the paralinguistic dynamics and emotional evolution throughout the consultation, we further implemented a temporal modeling architecture using a BiLSTM network. The sequence of segment embeddings is fed into the BiLSTM layers to extract dependencies from both forward and backward temporal directions, capturing critical nuances such as hesitation patterns or distress escalation. The final hidden states are aggregated via a pooling layer to generate a comprehensive temporal audio representation for downstream multi-label classification.”

7.Missing data in Table 1 (marked with *) must be handled explicitly. Provide denominators for each variable and state a defined missing-data strategy (complete-case vs imputation).

**Response:**

We appreciate the editor’s request for clarification regarding the missing data in our demographic analysis. We have clarified the handling of missing demographic data in the revised manuscript. We adopted a complete-case analysis strategy, where statistical counts for each variable were calculated based only on individuals with non-missing information. The specific denominators for the High-risk and Control groups across variables with missing values are as follows:

- **Age:** High-risk: $n=488$; Control: $n=489$; Total: $n=977$
- **Education Level:** High-risk: $n=375$; Control: $n=423$; Total: $n=798$
- **Occupation:** High-risk: $n=436$; Control: $n=458$; Total: $n=894$
- **Marital Status:** High-risk: $n=467$; Control: $n=477$; Total: $n=944$

We did not use imputation for these demographic factors, as they were used for population characterization rather than as primary features in our predictive models. Importantly, we would like to emphasize that the primary variables used for machine learning modeling and label generation—Mood status, Suicidal ideation, Suicidal plan, and High-risk status—had a 100% completion rate ($n=1,057$).

We have included the following text in the footnotes of Table 1 on Page 7:

“A complete-case analysis strategy was applied to these variables, with denominators as follows: Age (High-risk: *n*=488; Control: *n*=489; Total: *n*=977), Education level (High-risk: *n*=375; Control: *n*=423; Total: *n*=798), Occupation (High-risk: *n*=436; Control: *n*=458; Total: *n*=894), and Marital status (High-risk: *n*=467; Control: *n*=477; Total: *n*=944).”

8.Education stratification is not defined. “Low/medium/high” must be explicitly mapped to concrete education categories.

**Response:**

We appreciate the editor’s attention to detail regarding the demographic classifications. We would like to clarify that the explicit mapping for education levels was provided in the footnote of Table 1 in the original manuscript (Page 8). The stratification is defined as follows:

- **Low education:** Primary school or below.
- **Medium education:** High school or vocational school.
- **High education:** College or above.

9.Fix typos (including the “.Audio library” and reference), and revise ambiguous sentences in Methods/Results so that data flow, model inputs, and evaluation steps are unambiguous.

**Response:**

We greatly appreciate the editor’s keen observation of detail. We have thoroughly proofread the entire manuscript.

Reviewer #1:

This study analyzes psychological crises from the hotline by comparing audio-based solutions and text-based solutions. The study is comprehensive because it covers both audio-based and text-based approaches, and it evaluates both deep learning-based approaches and LLM-based approaches.

Some details of the study need to be further clarified.

Line 35: "Surprisingly, XXX, utilizing few-shot learning, exhibited the highest F1 scores across all multi-label tasks."

Why is this surprising, and how to explain this surprising result?

**Response:**

The result was considered “surprising” because traditional deep learning frameworks typically require extensive labeled datasets—in our case, hundreds of annotated calls from 2022—to effectively learn the nuances of psychological crisis detection. In contrast, DeepSeek-R1 and GPT-4o model achieved superior F1 scores using a few-shot learning strategy, which provided only two reference examples (one high-risk and one control) within the prompt.

We contribute this high performance to three primary factors:

1. **Massive Pre-trained Knowledge and Generalization:** Large Language Models (LLMs) are trained on vast text datasets with hundreds of billions of parameters, allowing them to internalize a deep understanding of language structures, emotional semantics, and human psychology long before encountering our specific dataset. This allows the model to generalize effectively from a few examples by leveraging its existing “mental health” knowledge base.
2. **Advanced Reasoning Capabilities:** Specifically, DeepSeek-R1 and GPT-4 utilize reinforcement learning to enhance their reasoning through “self-reflection” and “chain-of-thought” processes. This enables LLMs to interpret complex, non-linear crisis narratives—such as distinguishing between general distress and specific suicidal intent—more effectively than static embedding-based classifiers.
3. **In-Context Learning and Prompt Engineering:** By using carefully designed prompts that include Chain-of-Thought instructions, we guided the model to evaluate risk step-by-step (e.g., from ideation to planning). This structured reasoning allows the LLM to mimic the clinical judgment of a trained operator, achieving high-fidelity results without the need for traditional supervised weight updates.

We have included the following text in the Abstract (Page 2):

“Notably, GPT 4o and DeepSeek-R1, utilizing few-shot learning, demonstrated performance comparable to the GPT-embedding deep learning model across multiple tasks. This suggests that their advanced Chain-of-Thought reasoning effectively mitigates data dependency gap, enabling LLMs to align with clinical domains using a few examples.”

Line 119: How to define "high-risk calls"? Are they manually annotated?

**Response:**

Yes, the “high-risk” labels in our dataset were manually annotated by trained hotline operators based on the Depression and Suicide Risk Assessment questionnaire (S1 Table). The annotation process followed these steps:

1. **Professional Administration:** All calls were handled by operators who had undergone specialized suicide risk assessment training.
2. **Guided Exploration:** During the consultation, operators used the 20 items in S1 Table as a framework, probing deeper into relevant clinical dimensions (e.g., mood, hopelessness, or intent) as they naturally surfaced during the conversation.
3. **Post-call Labeling:** Immediately following the conclusion of each call, operators completed standardized electronic logs and assigned the four study labels (mood status, suicidal ideation, suicidal plan, and high-risk status) according to the explicit criteria listed in S1 Table. This ensures that the labels reflect the totality of the professional interaction and provide a reliable ground truth.

The definition of high risk was provided in the “Data collection and selection” section on Page 15:

High‑risk call evaluation criteria (for details, see S1 Table):

Definition of high-risk calls[37]: (1) Individuals with suicidal thoughts and specific suicide plans. (2) Individuals with suicidal thoughts but no plan, with a risk factor score≥5.

In addition, the emotional state of the callers is also considered as a risk factor in the scoring system (Moderate depression is assigned 1 point, and severe depression is assigned 2 points).

Line 201: It is interesting to see "multimodal synergy". It is further described in equation (5) on Line 504. However, there is no result for this experiment.

**Response:**

We thank the reviewer for pointing out this potential source of confusion. We would like to clarify that the results for the "multimodal synergy" experiment (described by the Attention-based Gated Fusion architecture in Equation (5) are indeed present in the manuscript, labeled as “Attention-based Fusion”.

Specifically:

Visual Results: The performance metrics for this fusion model are displayed in Figure 2 on Page 9 (labeled in the legend as “Attention-based Fusion (Whisper+GPT)”).

Textual Discussion: A detailed analysis of these results is provided in the Results section under the heading “Multidimensional prediction with crafted models”. We explicitly state there that “fusion experiments did not yield statistically significant performance gains over the unimodal GPT text baseline”.

Tabular Data: The comprehensive numerical performance indicators (Precision, Recall, F1-Score, and Accuracy) for this experiment are listed in S4 Table under the “Attention-based Fusion” label.

Figure 4: What are rubrics to evaluate label consistency, logical reasoning, information completeness, clinical relevance, and clarity and comprehensibility in the expert evaluation?

**Response:**

We thank the reviewer for this question. We would like to clarify that detailed descriptions of the rubrics for each evaluation dimension are provided in S1 Appendix of the Supplementary Material. To facilitate the review process, we have summarized the core criteria for each rubric below:

- **Consistency with Labeling:** Assesses whether the explanation accurately reflects the model's predicted labels (e.g., “depression” or “high risk”) and if it establishes a reasonable relationship between the call content and those labels.
- **Logic and Reasoning Plausibility:** Evaluates the internal logic of the explanation and whether the reasoning process clearly demonstrates how the final labels were derived from the caller's dialogue.
- **Completeness and Accuracy:** Checks for the inclusion of all critical information and the absence of factual errors or omissions regarding the caller's situation.
- **Clinical and Situational Relevance:** Determines the practical applicability of the explanation in a clinical setting and its ability to guide professionals in real-world decision-making.
- **Clarity and Comprehensibility:** Focuses on the linguistic quality—whether the text is clear, easy to understand, and can be quickly processed by a professional under pressure.

All dimensions were scored by two independent experts using a 5-point Likert scale.

We have added a reference to S1 Appendix in the caption of Figure 4 on Page 12 to ensure readers can easily locate these definitions.

"The specific scoring rubrics for these five dimensions, including detailed definitions and clinical criteria, are provided in S1 Appendix."

Line 472: "combining the reconstruction task of an autoencoder with the classification task" is described in S2. What is the purpose of using an auto-encoder and sharing it with the classification task, instead of using a classification framework only?

**Response:**

We thank the reviewer for this technical question. The decision to integrate an autoencoder (AE) into our classification framework was driven by the following primary objectives:

1. **Dimensionality Reduction and Latent Feature Extraction:** The pre-trained embeddings used in this study, particularly the GPT-based embeddings, have a high dimensionality of 3,072. An autoencoder is designed for unsupervised learning to map this high-dimensional input ($\epsilon$) into a lower-dimensional, compressed latent space ($z$). By sharing the encoder between the reconstruction and classification tasks, we ensure that the latent variable $z$ captures the most essential structural features of the raw data.
2. **Regularization through Joint Optimization:** A pure classification framework may overfit on high-dimensional noise, especially with a finite clinical dataset. By adding a reconstruction loss ($L_{rec}$), the model is forced to preserve the integrity of the original input while simultaneously performing the classification task ($L_{clf}$). This dual-task optimization ($L= \lambda_{1}\cdot L_{rec}+ \lambda_{2}\cdot L_{clf}$) acts as a regularizer, enhancing the model's stability and generalization ability.
3. **Maximizing Data Utilization for Multi-label Tasks:** Our task involves four interdependent binary labels (mood, ideation, plan, and high-risk). Using an autoencoder allows the model to leverage the underlying data distribution in an unsupervised manner (via reconstruction), which provides a more robust feature foundation for the supervised multi-label prediction task.

We have included the following text in the “Deep learning framework with pre-trained model” section on Page 17:

“This joint learning approach ensures that the extracted features are not only discriminative for classification but also representative of the original data structure, providing a regularization effect that prevents the model from overfitting on high-dimensional noise.”

The whole manuscript, please change the reference citation from () to [].

**Response:**

All references have been converted from rounded brackets () to square brackets [] throughout the manuscript.

Reviewer #2:

This study presents a rigorous evaluation of pre-trained deep learning models and large language models (LLMs) for multi-label crisis detection in psychological support hotlines. Using 1,057 real-world hotline calls, the authors demonstrate that text-based semantic representations—particularly GPT-derived embeddings—significantly outperform acoustic-only and multimodal approaches in identifying emotional distress and suicide risk, achieving an F1 score of 80.48% for high-risk detection. Notably, the work shows that few-shot LLMs (DeepSeek-R1) can match supervised deep learning performance while requiring minimal labeled data. The integration of expert evaluation of model-generated explanations adds important clinical relevance and interpretability.

Action item:

Validate models on multicenter, culturally diverse hotline datasets to improve generalizability.

**Response:**

We thank the reviewer for highlighting the importance of cross-cultural validation. We acknowledge that the generalizability of our crisis detection framework to diverse populations is a critical factor for its broader implementation. To address this, we conducted a cross-linguistic transfer experiment using the Distress Analysis Interview Corpus (DAIC, *Gratch J, Artstein R, Lucas GM, Stratou G, Scherer S, Nazarian A, et al. The distress analysis interview corpus of human and computer interviews. Lrec, vol. 14, Reykjavik; 2014, p. 3123–8*).

**Benchmark Dataset:** Because psychological support hotline datasets are typically not publicly available due to strict privacy and confidentiality protections, we utilized the DAIC as a representative, culturally diverse English-language benchmark. The DAIC consists of semi-structured clinical interviews conducted in Los Angeles, USA, involving a diverse population of both veterans and the general public. The dataset used in our validation included 189 participants (57 depressed and 132 non-depressed), with labels derived from the Patient Health Questionnaire (PHQ-8), depression module.

**Cross-Linguistic Transfer Methodology:** In our original study, the neural network model utilizing GPT embeddings (text-embedding-3-large) achieved the highest performance across all tasks. This specific model is natively multilingual. To test its generalizability, we applied it to the participants' transcribed speech in the DAIC dataset to generate 3,072-dimensional embeddings.

**Validation Results:** Given the smaller sample size of the DAIC, we employed Principal Component Analysis (PCA) to reduce the embeddings to 60 dimensions and trained a Support Vector Machine (SVM) classifier to differentiate between depressed and non-depressed individuals. The results of this experiment (illustrated in the confusion matrix in the Fig S8) demonstrate robust transfer performance:

- **Precision:** 62.50%
- **Recall**: 71.43%
- **F1-Score:** 66.67%
- **Accuracy:** 78.72%

**Conclusion:** The fact that our framework—without any language-specific fine-tuning—achieved over 78% accuracy on an English-speaking, culturally distinct population provides strong evidence of its potential for global generalizability. These results confirm that the GPT-embedding-based model is sensitive to core linguistic indicators of psychological distress that transcend cultural and language barriers.

Comprehensive details regarding the data characteristics and the classification performance of this cross-linguistic validation are illustrated in the added S8 Fig of the Supplementary Material.

We have included the following text in the Discussion section on Page 13:

“Furthermore, we extended our evaluation to the Distress Analysis Interview Corpus (DAIC) [36] to assess the framework’s cross-cultural robustness. By extracting GPT-embeddings from DAIC transcripts, a simple SVM classifier achieved a classification accuracy of 78.72% and a recall of 71.43% for depression detection (S8 Fig). These preliminary results indicate that GPT embedding model successfully captures universal linguistic markers of psychological distress within a culturally diverse population.”

Address class imbalance reflective of real-world call distributions.

**Response:**

We completely agree that evaluating the model under a distribution that reflects real-world prevalence is essential for assessing its practical utility. To address this, we conducted a prevalence-aware simulation based on the actual call volume of the Hangzhou Hotline in 2023. In 2023, the Hangzhou Hotline received a total of 18,245 calls, of which 276 were clinically identified as high-risk (a prevalence of approximately 1.5%). To simulate this imbalance, we re-evaluated our best-performing model (the GPT-embedding neural network) by randomly sampling calls from our prospective test set to reflect a ratio of 4 high-risk calls to 270 controls.

The results of this prevalence-aware evaluation (see S7 table in the Supplementary Material) demonstrate the following:

1. **Impact on Precision and F1-Score:** As expected in a low-prevalence scenario, the Precision and F1-score decreased across all four tasks. This is a common mathematical artifact when the “true positive” pool is extremely small compared to the “true negative” pool.
2. **Robustness of Recall and Accuracy:** For the “High Risk vs. Non-High Risk” classification task, the model maintained a Recall of 78.75% [74.50%, 82.76%] and a Balanced-Accuracy of 78.67% [76.59%, 80.70%].
3. **Clinical Utility:** In the context of psychological crisis intervention, prioritizing Recall is paramount to ensure that high-risk individuals are not missed (minimizing Type II errors). A recall of nearly 79% in a simulated real-world environment suggests that the model can effectively serve as a “safety net” or screening tool to alert human operators to potential crises that require immediate attention.

The comprehensive performance metrics for all evaluated models under these simulated real-world conditions are presented in the added S7 Table of the Supplementary Material.

We have included the following text in the “Multidimensional prediction with crafted models” section on Page 8:

“To evaluate model’s generalizability to real-world settings where high-risk calls are infrequent, we performed a prevalence-aware simulation. We simulated a 1.5% high-risk prevalence—mirroring 2023 hotline statistics—with a 4:270 high-risk-to-control ratio. Comprehensive performance metrics for each method under these imbalanced conditions are provided in S7 Table. We observed that while precision naturally declined due to low prevalence, GPT embedding still maintained high recall (78.75% [74.50%, 82.76%]) and accuracy (78.67% [76.59%, 80.70%]) in the High Risk vs. Non-High Risk classification task. This suggests the model remains an effective decision-support tool for prioritizing urgent interventions even in imbalanced conditions.”

We have included the following text in the Discussion section on Page 13:

“While our study utilized a matched case-control design to optimize feature learning, this may not fully reflect model performance in real-world settings where high-risk callers represent a small minority (approximately 1.5%). Nevertheless, our prevalence-aware simulation demonstrates that the GPT-embedding deep learning model remains a robust screening instrument. Although its Positive Predictive Value (PPV) is sensitive to such skewed distributions, the model maintains a high recall. In the high-stakes context of psychological crisis intervention, prioritizing recall is paramount to ensuring that no high-risk individual is overlooked. The high sensitivity of this framework suggests its potential to function as an automated “safety net”, effectively alerting operators to latent crises that necessitate immediate clinical attention.”

We have included the following text in the Discussion section on Page 14:

“Third, while our prevalence-aware simulation validated the GPT-embedding deep learning model as a high-recall screening tool, its PPV remains sensitive to the low-prevalence typical of real-world settings. Future deployment will necessitate cost-sensitive learning or advanced data augmentation to manage extreme class imbalances without compromising clinical utility.”

Incorporate sequential or temporal fusion methods for richer audio modeling.

**Response:**

We sincerely thank the reviewer for this constructive suggestion. We agree that our initial approach—averaging Whisper/Wav2Vec embeddings over 10-second segments —may have collapsed important temporal dynamics such as hesitation patterns or distress escalation that are critical for crisis detection.

To address this, we conducted a supplementary comparative analysis by implementing a BiLSTM (Bidirectional Long Short-Term Memory) architecture. This model variant was designed specifically to preserve and model the sequential dependencies within the Whisper-derived embeddings, rather than treating them as static averages.

As shown in the added performance table (Table S5):

1. **Significant Recall Gains:** The BiLSTM model achieved a substantial increase in Recall across all categories. For instance, the Recall for the “High Risk vs. Non-High Risk” classification improved from 67.42% [64.83%, 70.02%] to 85.50% [84.15%, 86.82%].
2. **F1-Score and Reliability:** We observed a clear improvement in F1-scores, particularly in identifying “Suicidal plans”, which rose from 39.27% [36.99%, 41.51%] to 53.36% [52.50%, 54.18%]. This confirms that temporal modeling indeed captures a stronger acoustic signal than simple pooling.
3. **Modality Comparison:** However, even with this enhanced temporal modeling, the audio-only performance remained significantly lower than the text-only GPT-embedding framework. For the “High Risk vs. Non-High Risk” classification, the text-based model’s F1-score (80.48% [80.18%, 80.78%]) still substantially outperforms the best audio-only result (71.00% [70.69%, 71.31%]).

These additional experiments demonstrate that while acoustic temporal dynamics provide valuable information, the semantic content encoded in the text remains the most robust and predictive signal for crisis detection in this hotline context.

The comprehensive performance metrics for auditory temporal modeling across various pre-trained models are presented in the added S5 Table of the Supplementary Material.

We have included the following text in the “Multidimensional prediction with crafted models” section on Page 8:

“By adopting Bidirectional Long Short-Term Memory (BiLSTM) strategies to preserve temporal dynamics, we observed a significant improvement in recall for auditory models; however, the corresponding increase in F1-score remained modest (see S5 Table).”

We have included the following text in the Discussion section on Page 12:

“While adopting BiLSTM strategies to preserve time-series information significantly improved acoustic-based recall, the overall performance ceiling remained firmly dictated by the text modality.”

We have included the following text in the Discussion section on Page 14:

“Additionally, audio-text fusion experiments did not yield statistically significant performance improvements. Future work should move beyond modality-specific temporal modeling toward fine-grained, cross-modal attention mechanisms. Such strategies could enable the model to dynamically align transient acoustic markers with specific high-risk linguistic segments, thereby enhancing the synergy between vocal urgency and articulated intent.”

We have included the following text in the “Deep learning prediction framework with pre-trained model” section on Page 16:

“First, an averaging operation was applied to these embeddings to form a global representation of the call. However, to better capture the paralinguistic dynamics and emotional evolution throughout the consultation, we further implemented a temporal modeling architecture using a BiLSTM network. The sequence of segment embeddings is fed into the BiLSTM layers to extract dependencies from both forward and backward temporal directions, capturing critical nuances such as hesitation patterns or distress escalation. The final hidden states are aggregated via a pooling layer to generate a comprehensive temporal audio representation for downstream multi-label classification.”

Develop standardized, objective frameworks for evaluating LLM-generated clinical explanations.

**Response:**

We thank the reviewer for this constructive comment. We agree that developing standardized and objective frameworks is paramount to ensuring the clinical safety and reliability of LLM-generated outputs.

Our study implemented a five-dimensional evaluation framework designed to capture the clinical nuances of hotline crisis detection while maintaining objective rigor. This framework was developed by adapting established evaluation axes from state-of-the-art benchmarks for clinical LLMs, notably the human evaluation criteria used in the validation of Med-PaLM (*Singhal K, Azizi S, Tu T, Mahdavi SS, Wei J, Chung HW, et al. Large language models encode clinical knowledge. Nature 2023;620:172–80*). The dimensions include:

- **Consistency with labeling:** Verifies if the reasoning supports the predicted risk category, ensuring clinical consistency.
- **Logic and reasoning plausibility:** Evaluates the internal coherence of the reasoning process to prevent logical hallucinations.
- **Completeness and accuracy:** Directly mirrors standard axes to ensure factual integrity and capture all critical indicators.
- **Clinical and situational relevance:** Focuses on actionable utility for operators in urgent, real-world scenarios.
- **Clarity and comprehensibility:** Assesses the practical interpretability of the text under pressure.

To ensure objectivity, independent experts scored these dimensions on a 5-point Likert scale using a standardized rubric provided in S1 Appendix.

We have included the following text in the Discussion section on Page 14:

“A critical long-term goal for this field is the development of broader, automated, and standardized objective benchmarks. Such standardized frameworks are essential to move beyond subjective expert reviews and will be instrumental in further enhancing the clinical safety, transparency, and overall trustworthiness of LLM-generated insights in mental health services.”
